# Supplementary material for: ICOS Coreceptor Signaling Inactivates the Transcription Factor FOXO1 to Promote Tfh Cell Differentiation
Source: Immunity. 2015 Feb 17;42(2):239–51. doi: 10.1016/j.immuni.2015.01.017 (PMC4334393; doi:10.1016/j.immuni.2015.01.017)
Supplement: Document S1. Figures S1–S5 and Supplemental Experimental Procedures [file mmc1.pdf]

Immunity

Supplemental Information

ICOS Coreceptor Signaling Inactivates the Transcription Factor FOXO1 to Promote Tfh Cell Differentiation

Erica L. Stone, Marion Pepper, Carol D. Katayama, Yann M. Kerdiles, Chen-Yen Lai, Elizabeth Emslie, Yin C. Lin, Edward Yang, Ananda W. Goldrath, Ming O. Li, Doreen A. Cantrell, Stephen M. Hedrick

Figure S1; Stone et al.

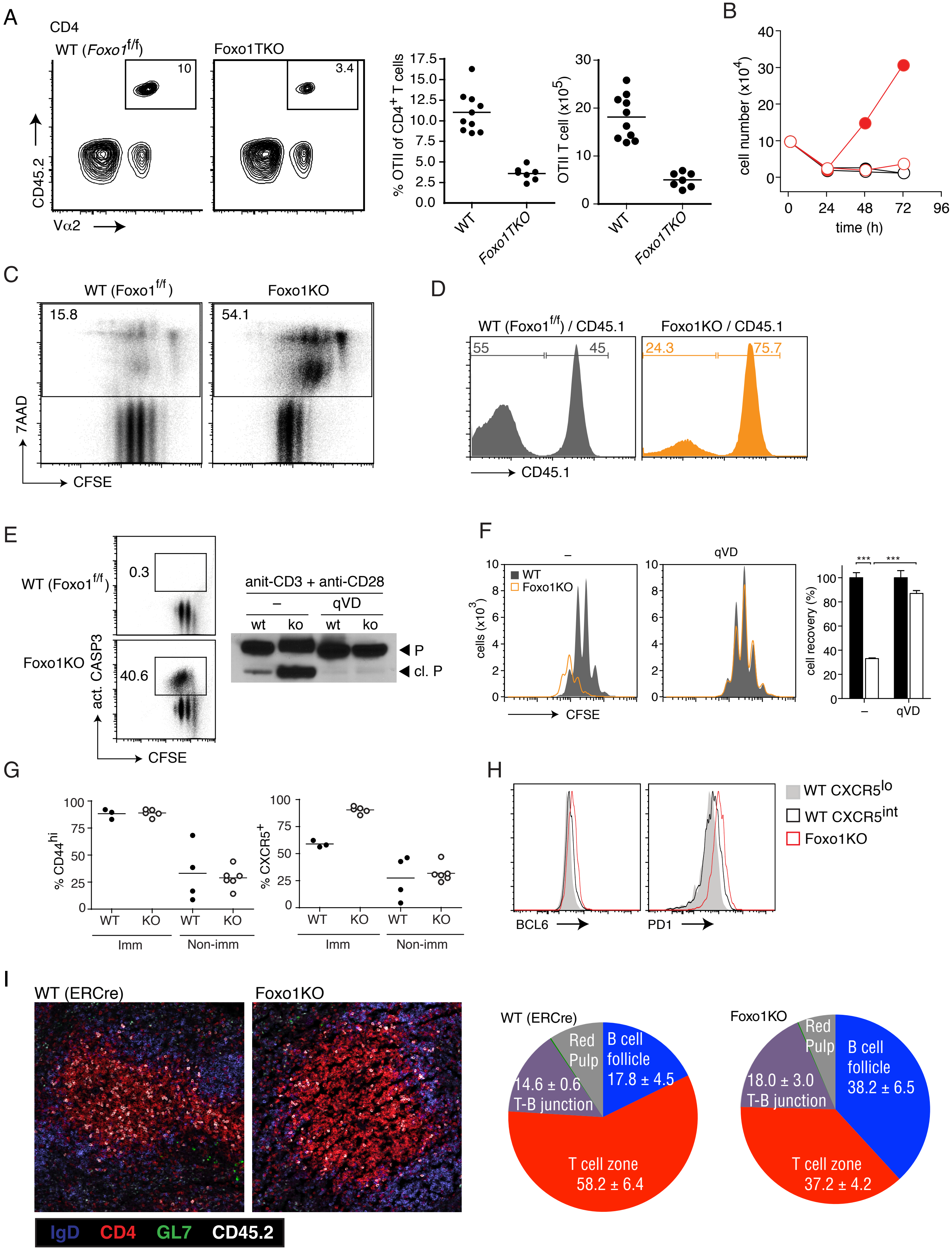

Figure S2, Stone et al.

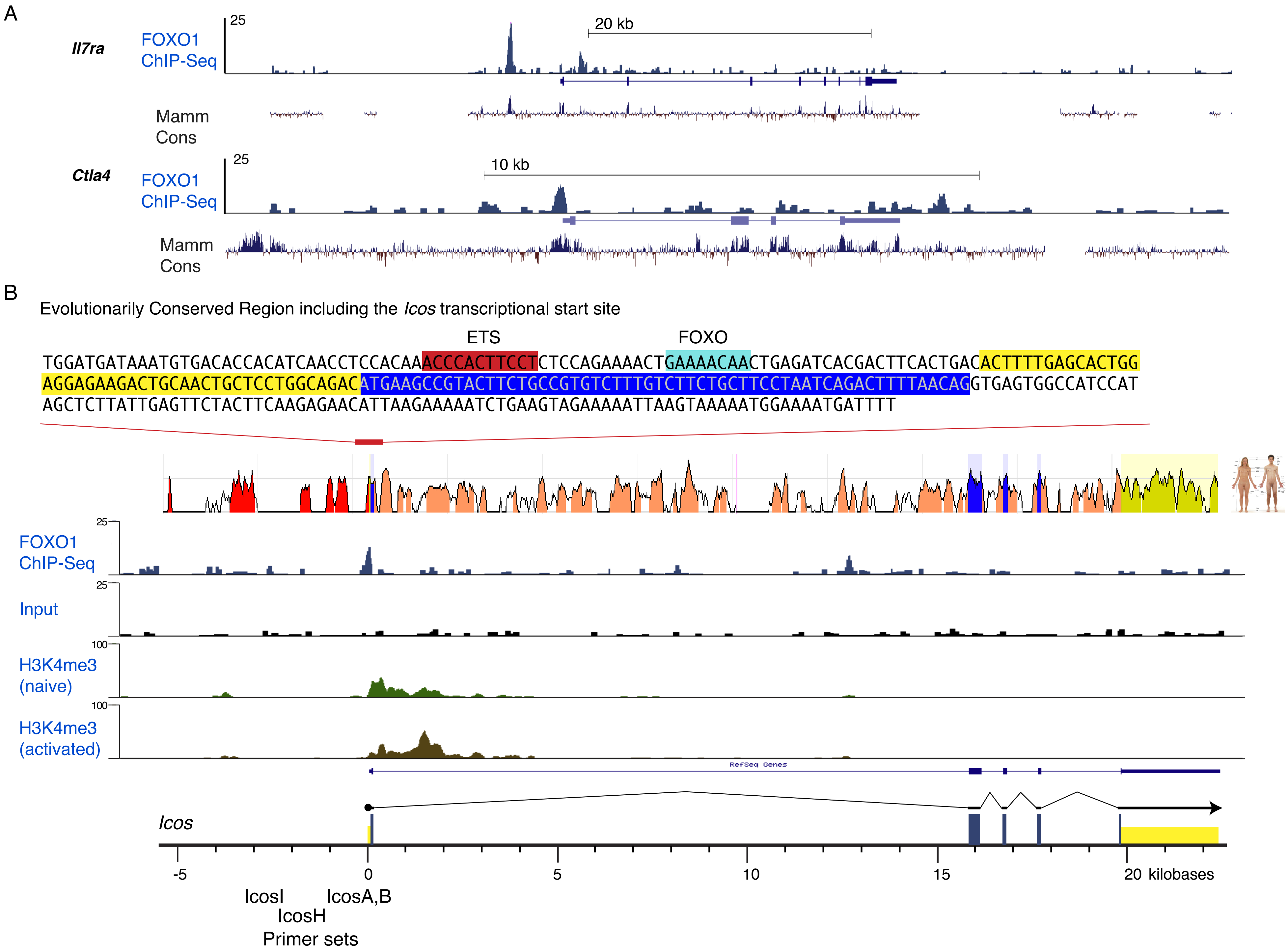

Figure S3, Stone et al.

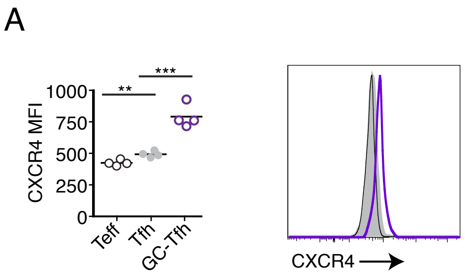

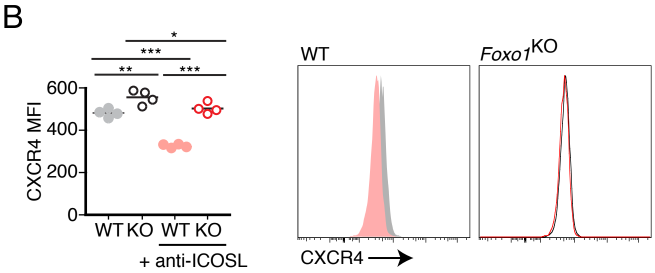

Figure S4, Stone et al.

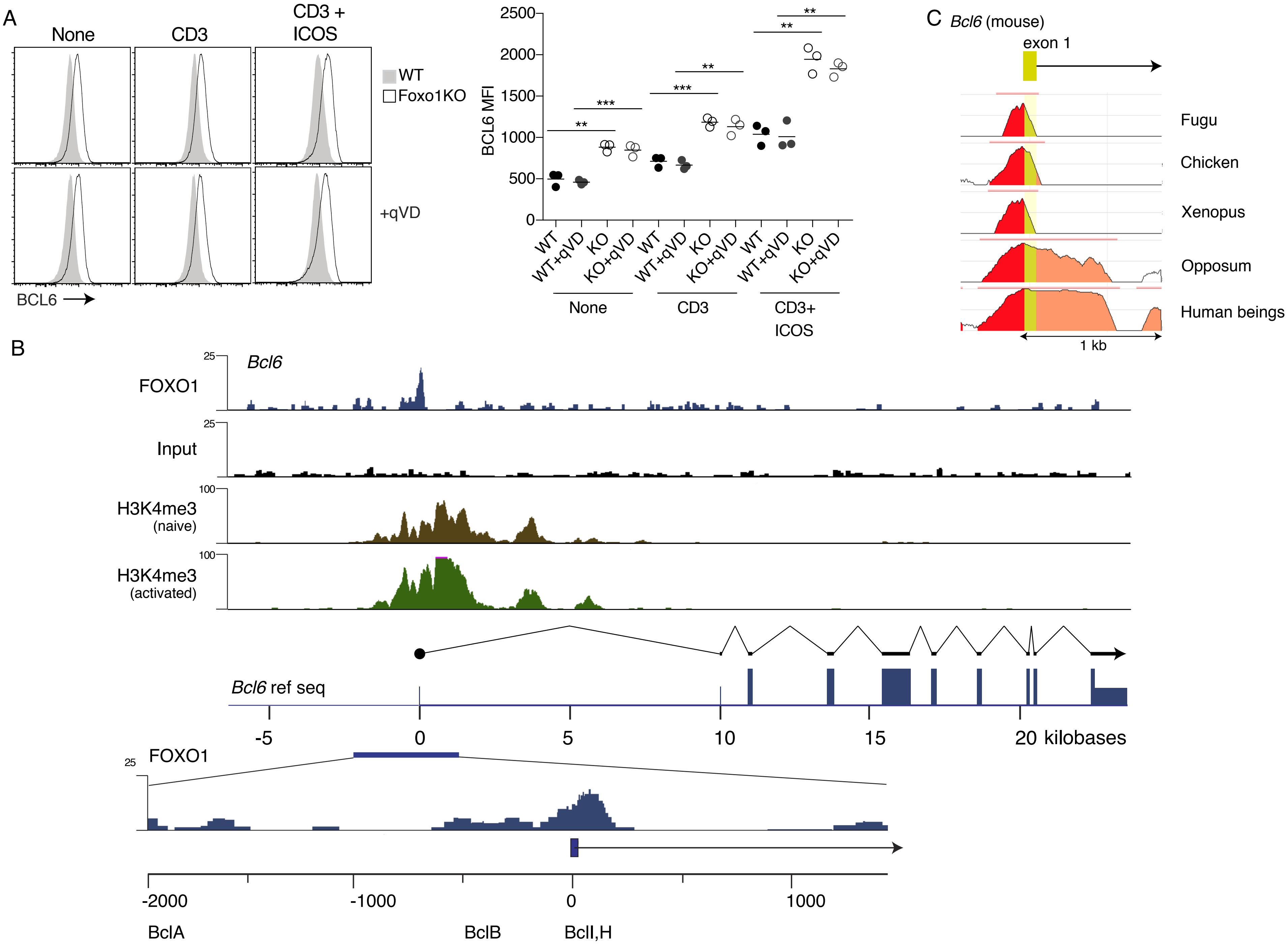

Figure S5, Stone et al.

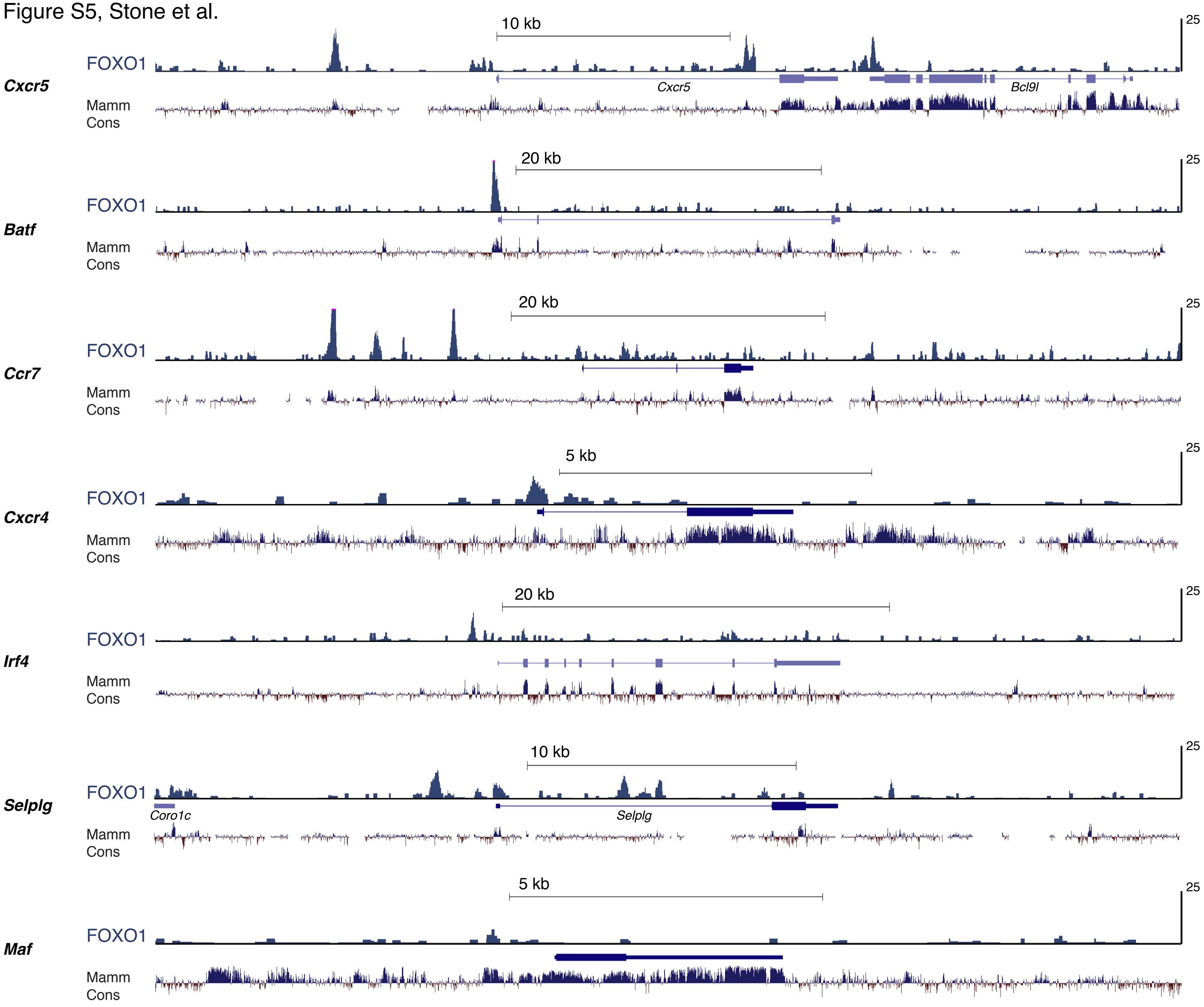

SUPPLEMENTAL FIGURE LEGENDS

Figure S1, related to Figure 1. FOXO1 is required to prevent activated CD4 T cell death

(A) Decreased accummulation of *Foxo1*^{TKO} T cells *in vivo*. OTII T cells were transferred into CD45.1 hosts, and immunized with OVA and adjuvant. At d 4, WT or *Foxo1*^{TKO} OTII CD4+ cells were analyzed for accumulation by gating for CD45.2^{+}Vα2^{+} T cells. Each dot represents a single host mouse. One of two representative experiments.

(B-F) Activated CD4 *Foxo1*^{KO} T cells die by apoptosis. LN naïve CD4 T cells (CD4^{+} CD69^{-} CD25^{-}) were isolated from tamoxifen-treated WT (*Foxo1*$^{f/f}$, filled circles) and *Foxo1*^{KO} (open circles) and tested as shown. (B) Decreased accumulation of *Foxo1*^{KO} CD4 T cells *in vitro*. Enumeration of purified naïve LN CD4 T cells stimulated with anti-CD3 in presence (red circles) or absence (black circles) of soluble anti-CD28 (mean + s.d. of triplicate cultures, one representative experiment out of three). (C) *Foxo1*^{KO} CD4 T cells progress normally through cell division but die at a high rate in the first rounds of division. Representative FACS profile of CFSE dilution vs. 7AAD from naïve LN CD4 T cells stimulated for 2 days with anti-CD3 and anti-CD28 (one representative experiment out of three). (D) Loss of *Foxo1*^{KO} CD4 T cells is cell-intrinsic. Relative cell recovery of anti-CD3 plus anti-CD28 stimulated naïve LN CD4 T cells co-cultured with naïve LN CD4 T cells from WT CD45.1 mice for 3 days (one representative experiment out of two). (E) Stimulation of *Foxo1*^{KO} CD4 T cells provokes active Caspase 3. Representative FACS profile of naïve LN CD4 T cells stimulated for 2 d with anti-CD3/anti-CD28 (one representative experiment out of two) (Left). Activated *Foxo1*^{KO} CD4 T cells display cleaved PARP. Western blot analysis of PARP cleavage in naïve LN CD4 T cells stimulated for 2 days with anti-CD3 plus anti-CD28 in the presence or absence of the pan-caspase inhibitor Q-VD-OPH (qVD) (one representative experiment out of two) (Right). (F) qVD rescues the accummulation of *Foxo1*^{KO} CD4 T cells. Naïve, CFSE-labeled LN CD4 T cells were stimulated for 3 days with anti-CD3 plus anti-CD28 in the presence or absence of the pan-caspase inhibitor qVD (one representative experiment out of two). The results were expressed as cell recovery from a timed-acquisition, such that the area under each curve is representative of the total accumulation of cells (mean + s.d.). Data is from experimental replicates.

(G) Activation and presence of Tfh phenotype in *Foxo1*^{KO} CD4 T cells is antigen dependent. Graphs show percentage of CD44^{hi} (Left) or CXCR5^{+} (Right) of CD4^{+}CD45.2^{+}Vα2^{+} WT or *Foxo1*^{KO} cells 4 days post immunization or non-immunized controls. (H) Total *Foxo1*^{KO} CD4 T cells express higher levels of Tfh markers than WT Tfh cells. Histograms show expression of BCL6 or PD1 d 4 post immunization within the WT (CD4^{+}CD45.2^{+}Vα2^{+}) CXCR5^{lo} or CXCR5^{int} population along with the total *Foxo1*^{KO} (CD4^{+}CD45.2^{+}Vα2^{+}) population for comparison. (I) T cells lacking FOXO1 localize to the follicular border. Immunofluorescence of spleens d 4 post immunization (Left). Pie charts show proportion of OTII cells (mean percentage +/- s.e.m.) located in each region determined by counting

one representative image in a blinded fashion from each of four mice per a donor genotype pooled from multiple experiments (Right). P-value is less than 0.05 between WT and *Foxo1*^{KO} in the T cell zone and between WT and *Foxo1*^{KO} in the B cell follicle.

Figure S2, related to Figure 2. Evolutionarily conserved FOXO1 binding sites within the *Il7ra, Ctla4* and *Icos* loci

(A) We have previously shown by ChIP that FOXO1 binds to an enhancer 3.3 kb upstream of the *Il7ra* TSS and within the promoter of the *Ctla4* gene (Kerdiles et al., 2009; Kerdiles et al., 2010). FOXO1 binding to the *Il7ra* and *Ctla4* locus as determined by ChIPSeq on naïve T cells is shown. (B) The *Icos* locus. Evolutionarily Conserved Regions (ECRs) in the mouse *Icos* gene were compared with human *ICOS*. The Ref Seq data was used to generate a map of mouse *Icos*, and the ECRs taken from decode.org are shown for comparison. Red ECRs are intergenic, yellow indicates untranslated transcriptional regions, and blue denotes exons. The ECR flanking the transcriptional start site was exploded, and it was predicted to have consensus ETS (red) and FOXO (cyan) binding sites. The approximate locations of four primer sets used for ChIP are shown (A and B are overlapping). FOXO1 ChIP-seq analysis at *Icos* locus is reshown from Figure 2F for comparison. Primer sequences are as follows: icosA for:CATACATCACCGGGTACTTGC, icosA rev:GCAGTCTTCTCCTCCAGTGC, icosB for:CCATTCATACATCACCGGGTA, icosB rev:CCAGGAGCAGTTGCAGTCTT, icosH for:GAGGCCAGAAGAGGACACTG, icosH rev:GCATGCCTGTAACCACAGAA, icosI for:AAGCATCCACCCTACAAACG, icosI rev:TGGATGTGAGAATGGAAGGA.

Figure S3, related to Figure 3. *Foxo1*^{KO} OTII cells are less dependent on ICOS for CXCR4 upregulation

Graphs show CXCR4 MFI d 4 post immunization of (A) WT CXCR5^{lo}, CXCR5^{int}, or CXCR5^{hi} OTII cells or (B) total WT or *Foxo1*^{KO} OTII cells from host mice treated with anti-ICOSL or isotype control. Data is from one experiment with at least four host mice per a genotype and condition.

Figure S4, related to Figure 5. Evolutionarily conserved FOXO1 binding site at the *Bcl6* locus

(A) WT (*Foxo1*$^{f/f}$) or *Foxo1*^{KO} naïve CD4 cells were activated as in Figure 5A in the presence or absence of the pan-caspase inhibitor Q-VD-OH (qVD) and BCL6 expression was determined by flow cytometry. Data is representative of one experiment with three mice per a genotype. (B) The *Bcl6* locus. ChIP-Seq data from the Bcl6 locus is shown for FOXO1, and H3K4m3 marks on both naïve and activated T cells. FOXO1 ChIP-Seq analysis at *Bcl6* locus is reshown from Figure 5D for comparison. The Ref Seq data was used to generate a map of mouse *Bcl6*. And the region surrounding the transcription start site is exploded to pinpoint the FOXO1 binding peak. The

approximate locations of four primer sets used for ChIP are shown. BclA is a negative control at approximately -2 kbp, and BclB corresponds to a FOXO1 binding site previously identified (Oestreich et al., 2012). Primer sequences are as follows: bclA for:GTACTCCAACAACAGCACAGC, bclA rev:GTGGCTCGTTAAATCACAGAGG, bclH for:GAGCAATGGTAAAGCCCG, bclH rev:CAACAGCAATAATCACCTGG, bclI for:CGCTGCTCATGATCATTAT, bclI rev:GTATGCGAAAAGCTAGATCCT, IL7Ra for:ACCTCATCAGCCTTTCATGG, IL7Ra rev:ATCCCCTGAGCAAACTAGCA.(C) *Bcl6* ECRs within the region surrounding the first exon compared with: Human beings, Opposum, Xenopus, Chicken, and Fugu. The pink line above each element of the figure indicates conservation of at least 100 bp with 70% or more similarity. We note the extraordinary conservation of the first non-coding exon and the 5' end of the first intron.

Figure S5, related to Figure 6. ChIP-seq reveals FOXO1 binding to key Tfh genes.

The *Cxcr5*, *Batf*, *Ccr7*, *Cxcr4*, *Irf4*, *Selplg* and *Maf* loci are shown for FOXO1-specific ChIP-Seq and mammalian sequence conservation (UCSC genome browser).

SUPPLEMENTAL EXPERIMENTAL PROCEDURES, related to Experimental procedures.

Flow cytometry Generation of FOXO1-EGFP knock-in mice

The FOXO1-EGFP knock-in mice were generated at Taconic. Briefly, the *EGFP* sequence was inserted between the codon coding for the last amino acid of FOXO1 and the termination codon in exon 2 of the *Foxo1* gene which allows for translation of a FOXO1-EGFP fusion protein. Furthermore, exon 2 including the *EGFP* sequence was flanked by *loxP* sites allowing conditional deletion. The targeting vector included *FRT* sites to allow for FLP-mediated removal of *Neo*^{r}. The targeting vector was transfected into TaconicArtemis C57BL/6N Tac ES cells and selected for homologous recombination. FLP-mediated removal of selection markers was accomplished by breeding to mice expressing a *Flp transgene*.

Flow cytometry

Fluorochrome-conjugated antibodies for flow cytometry were from BD Biosciences (San Diego, CA), Biolegend (San Diego, CA) or eBioscience (San Diego, CA) unless otherwise indicated. For CXCR5 a teritary staining protocol was used. Rat anti-mouse CXCR5 (Clone 2G8; BD Biosciences) was allowed to bind for 1h at 4 degrees, biotin-labeled goat anti-rat IgG (Jackson ImmunoResearch) was used as the secondary and allowed to bind for 30 min at 4°C. Finally, fluorochrome-conjugated strepavidin was added with a cocktail of the remaining extracellular antibodies. Cells were fixed and permeabilized using the Foxp3 Permeabilization/Fixation kit (eBioscience, San Diego, CA). FOXO1 intracellular staining was done as previously described (Kerdiles et al., 2010). Cells were analyzed using LSR Fortessa or Calibur flow cytometers (Becton Dickinson) unless otherwise indicated. Traditional flow cytometry data were analyzed with FlowJo software (TreeStar).

Immunofluorescence

Spleen sections were prepared for immunofluorescence as previously described (Cheung et al., 2009). Briefly, freshly harvested tissues were fixed with 4% formaldehyde and soaked in sucrose overnight before embedding in OCT and freezing with dry ice. Six-micrometer-thick tissue sections were cut and fixed in paraformaldehyde. Sections were blocked in a solution of 10% BSA, 2.5%

normal goat serum, 2.5% normal donkey serum, and fish scale gelatin. Tissue sections were then incubated with combinations of conjugated or biotinylated antibodies. Sections were mounted using Invitrogen ProLong Gold antifade reagent. Images were taken with an Olympus FV1000 confocal microscope with five laser lines at wavelengths of 405, 458, 488, 515, 543, and 647 nm, using 10× and 20× air objectives. Images were analyzed using ImageJ. One representative image from each spleen was blinded and the proportion of $CD45.2^+$ cells in each region of the spleen was determined. For this images were blown up, a grid was placed over the image and then for every $CD45.2^+$ cell in the image it was determined by eye if the cell was located in the T cell area, Follicle, T/B boundry, GC or other area. This was done in a blinded fashion.

Retroviral transduction experiments

Retrovirus was generated by transducing plat-E cells with the pHR-MMPCreGFP retroviral vector (Silver and Livingston, 2001). Retroviral transductions were done as previously described with slight modifications (Johnston et al., 2009). Briefly, naive WT or *Foxo1*^{AAA} *OTII* cells were purified and activated with plate bound anti-CD3 and soluble anti-CD28 for 24 h. Cells were transduced with the resulting retrovirus at 24 h and 48 h. Cells were cultured with plate bound anti-CD3 and soluble anti-CD28 and IL-2 from 24 h to 72 h. After 72 h cells were cultured in media supplemented with IL-2 for an additional 3 days after which they were transferred into CD45.1 hosts. Hosts were immunized with OVA plus adjuvant as above 3 days after adoptive transfer.

In vitro death and proliferation experiments

Naive CD4 T cells were isolated by magnetic depletion of cells labeled with biotinylated antibodies to TER119, B220, MHCII, DX5, CD8, CD11B, CD25, and CD69 (eBioscience) and streptavidin-microbeads (Miltenyi Biotec). Where indicated, purified T cells were labeled with CFSE as previously described (D'Souza et al., 2008). To activate T cells, 1 x 10^5 cells/well were cultured in U bottom 96 well plates. Plates were coated with goat anti-Hamster IgG (Vector Labs) followed by anti-CD3 (2C11). Where indicated, cultures were supplemented with soluble anti-CD28 (Biolegend) or 20 μM pan-caspase inhibitor qVD (SM Biochemicals).

ChIP and ChIP-seq

Naïve CD4 T cells were purified as described above. Where indicated 5 x 10^5 cells per a well were activated in 24 well plates coated with anti-CD3 in the presence of anti-CD28 for 72 h. In other experiments cells were stimulated under iTfh conditions. FOXO1 ChIP-seq was done as previously described with minor modifications (Lin et al., 2010). Briefly, 3 x 10^7 T cells were fixed for 5 to 10 minutes at room temperature in 1% formaldehyde then resuspended in lysis buffer. Chromatin was sonicated to an average size of 400-500 base pairs and immunoprecipitated with 8 μg of anti-FOXO1 (clone: H-128, Santa Cruz Biotechnology) or anti-trimethyl-Histone H3 Lys4 (EMD Millipore). Bound chromatin was collected using protein G sepharose and de-crosslinked overnight at 65° C. Following RNaseA and proteinase K treatment, DNA was purified using a Qiaquick PCR Purification kit (Qiagen). For ChIP analysis, QPCR was performed using Fast Start Universal Syber Green master mix from Roche and analyzed on a Stratagene MX3005P real time thermal cycler. Homogeneous PCR products were validated by melting curve analyses.

For ChIP-seq samples were prepared for multiplex sequencing as recommended by Illumina. Following adaptor ligation, the DNAs were size selected (200-300bp) by 8% PAGE and index primers added by PCR. Samples were finally purified by 8% PAGE and precipitated with ethanol. Sequencing was performed by the BIOGEM core at UC San Diego using an Illumina HiSeq 2000 sequencer (50 cycles). Sequences were mapped to the mouse genome mm9 assembly (NCBI) using the Bowtie alignment tool and unique tags were visualized by preparing custom tracks for the UCSC Genome Browser where the total number of tags was normalized to 1 x 10^7. DNA sequence analysis was performed using HOMER software and instructions for analysis can be found at http://biowhat.ucsd.edu/homer/. The mammalian sequence conservation tracks were taken from the UCSC genome browser using the 30-Way Multiz Alignment and Conservation routine. Data has been deposited in the GEO database (Hess Michelini et al., 2013).

ELISA

Mice were bled at 4-6 months of age and anti-dsDNA antibodies of the IgG isotype in sera were determined by anti-mouse dsDNA IgG-specific ELISA kit (Alpha Diagnostic Int). Total sera IgG was

determined using Mouse IgG total Ready-Set-Go! ELISA (eBioscience) as per manufactures instructions.

SUPPLEMENTAL REFERENCES, related to Experimental procedures

Cheung, K.P., Yang, E., and Goldrath, A.W. (2009). Memory-like CD8+ T cells generated during homeostatic proliferation defer to antigen-experienced memory cells. J Immunol *183*, 3364–3372.

D'Souza, W.N., Chang, C.F., Fischer, A.M., Li, M., and Hedrick, S.M. (2008). The Erk2 MAPK regulates CD8 T cell proliferation and survival. J Immunol *181*, 7617–7629.

Johnston, R.J., Poholek, A.C., DiToro, D., Yusuf, I., Eto, D., Barnett, B., Dent, A.L., Craft, J., and Crotty, S. (2009). Bcl6 and Blimp-1 are reciprocal and antagonistic regulators of T follicular helper cell differentiation. Science *325*, 1006–1010.

Lin, Y.C., Jhunjhunwala, S., Benner, C., Heinz, S., Welinder, E., Mansson, R., Sigvardsson, M., Hagman, J., Espinoza, C.A., Dutkowski, J., Ideker, T., Glass, C.K., and Murre, C. (2010). A global network of transcription factors, involving E2A, EBF1 and Foxo1, that orchestrates B cell fate. Nat Immunol *11*, 635–643.
