# Supplementary material for: ICOS Coreceptor Signaling Inactivates the Transcription Factor FOXO1 to Promote Tfh Cell Differentiation
Source: Immunity. 2015 Feb 17;42(2):239–51. doi: 10.1016/j.immuni.2015.01.017 (PMC4334393; doi:10.1016/j.immuni.2015.01.017)
Supplement: Document S2. Article plus Supplemental Information [file mmc2.pdf]

Immunity

Article

ICOS Coreceptor Signaling Inactivates the Transcription Factor FOXO1 to Promote Tfh Cell Differentiation

Highlights

- ICOS signaling transiently inactivates FOXO1 to generate Tfh cells
- FOXO1 regulates a Tfh cell gene program exemplified by negative regulation of *Bcl6*
- Enforced nuclear localization of FOXO1 prevents Tfh cell differentiation
- FOXO1 promotes final GC-Tfh cell differentiation

Authors

Erica L. Stone, Marion Pepper, ..., Doreen A. Cantrell, Stephen M. Hedrick

Correspondence

shedrick@ucsd.edu

In Brief

How ICOS signaling causes the induction of BCL6 leading to Tfh cell differentiation is incompletely understood. Hedrick and colleagues show that ICOS signaling transiently inactivates FOXO1, which in turn relieves FOXO1-dependent inhibition of BCL6 expression and Tfh differentiation. In contrast, FOXO1 promotes late-stage germinal center-Tfh cell differentiation.

Stone et al., 2015, Immunity *42*, 239–251
February 17, 2015 ©2015 Elsevier Inc.
http://dx.doi.org/10.1016/j.immuni.2015.01.017

ICOS Coreceptor Signaling Inactivates the Transcription Factor FOXO1 to Promote Tfh Cell Differentiation

Erica L. Stone,[1] Marion Pepper,[2] Carol D. Katayama,[1] Yann M. Kerdiles,[1] Chen-Yen Lai,[1] Elizabeth Emslie,[3] Yin C. Lin,[4] Edward Yang,[4] Ananda W. Goldrath,[4] Ming O. Li,[5] Doreen A. Cantrell,[3] and Stephen M. Hedrick[1,4,*]
[1]Department of Cellular and Molecular Medicine, School of Medicine, University of California, San Diego, 9500 Gilman Dr., La Jolla, CA 92093-0377, USA
[2]Department of Immunology, University of Washington, 750 Republican Street, Seattle, WA 98109, USA
[3]College of Life Sciences, University of Dundee, Dow Street, Dundee, DD1 5EH, Scotland, UK
[4]Molecular Biology Section, Division of Biological Sciences, University of California San Diego, 9500 Gilman Dr., La Jolla, CA 92093-0377, USA
[5]Immunology Program, Memorial Sloan-Kettering Cancer Center, 1275 York Avenue, New York, NY 10065, USA
*Correspondence: shedrick@ucsd.edu
http://dx.doi.org/10.1016/j.immuni.2015.01.017

SUMMARY

T follicular helper (Tfh) cells are essential in the induction of high-affinity, class-switched antibodies. The differentiation of Tfh cells is a multi-step process that depends upon the co-receptor ICOS and the activation of phosphoinositide-3 kinase leading to the expression of key Tfh cell genes. We report that ICOS signaling inactivates the transcription factor FOXO1, and a *Foxo1* genetic deletion allowed for generation of Tfh cells with reduced dependence on ICOS ligand. Conversely, enforced nuclear localization of FOXO1 inhibited Tfh cell development even though ICOS was overexpressed. FOXO1 regulated Tfh cell differentiation through a broad program of gene expression exemplified by its negative regulation of *Bcl6*. Final differentiation to germinal center Tfh cells (GC-Tfh) was instead FOXO1 dependent as the *Foxo1*$^{-/-}$ GC-Tfh cell population was substantially reduced. We propose that ICOS signaling transiently inactivates FOXO1 to initiate a Tfh cell contingency that is completed in a FOXO1-dependent manner.

INTRODUCTION

The generation of high-affinity antibodies requires naive CD4$^+$ T cells to sequentially be activated, proliferate and differentiate, acquire proximity to the B cell follicles, and provide B cells with "help" in the form of antigen-specific interactions, co-receptor binding, and cytokine signaling. These specialized CD4 cells have been termed T follicular helper (Tfh) cells, and they are essential to promote the germinal center (GC) reaction including B cell expansion, class switching, selection, and development of high-affinity antibody-forming cells (Liu et al., 2013; Crotty, 2014; Ueno et al., 2015). In the past several years, much has been learned about Tfh cell differentiation; however, the cellular programming leading to this state remains incompletely understood.

Inducible T cell co-stimulator (ICOS) is a potent co-receptor distinct from CD28 that is induced on activated T cells and highly expressed on Tfh cells. ICOS signaling is necessary for complete GC development, T cell-dependent B cell help, and antibody class switching (Vinuesa et al., 2005), and this is due to a role for ICOS in the differentiation of activated T cells to Tfh cells (Ueno et al., 2015).

Tfh cell differentiation is a multi-step process that begins with dendritic cell priming and further requires B cells for additional differentiation and maintenance (Crotty, 2014; Ueno et al., 2015). The initial dendritic cell priming is sufficient to induce a CXCR5$^+$BCL6$^+$ Tfh cell, and this was found to be dependent on ICOS signaling (Qi et al., 2014). However, further ICOSL stimulation from B cells is required for the final differentiation and maintenance of GC-Tfh cells (Pepper et al., 2011; Crotty, 2014), and this is consistent with studies showing that ICOS is able to influence homing to GCs through the induction of filopodia (Franko and Levine, 2009; Xu et al., 2013). Signal transduction through ICOS results in the potent activation of phosphoinositide-3 kinase (PI3K), and this is a key event in Tfh differentiation (Rolf et al., 2010b). In a manner not yet understood, this leads to increased expression of BCL6, which has been described as an essential transcription factor for the differentiation and function of Tfh cells (Choi et al., 2013).

A major pathway downstream of PI3K signaling is the AKT-mediated inactivation of FOXO family transcription factors. AKT mediates the triple phosphorylation of FOXO proteins causing their nuclear egress (Calnan and Brunet, 2008). FOXO transcription factors are important for the expression of cyclin-dependent kinase inhibitors and proapoptotic molecules, and thus their inhibition is an essential aspect of growth factor-mediated cell-cycle progression and survival. In T cells, FOXO transcription factors have been shown to regulate multiple, specialized functions including the expression of the *Il7ra* and *Klf2*—control points for T cell survival and homing (Ouyang and Li, 2011; Hedrick et al., 2012). In addition, mice with a T cell-specific deletion of *Foxo1* lack functional FOXP3$^+$ Treg cells and spontaneously develop systemic autoimmunity. We previously

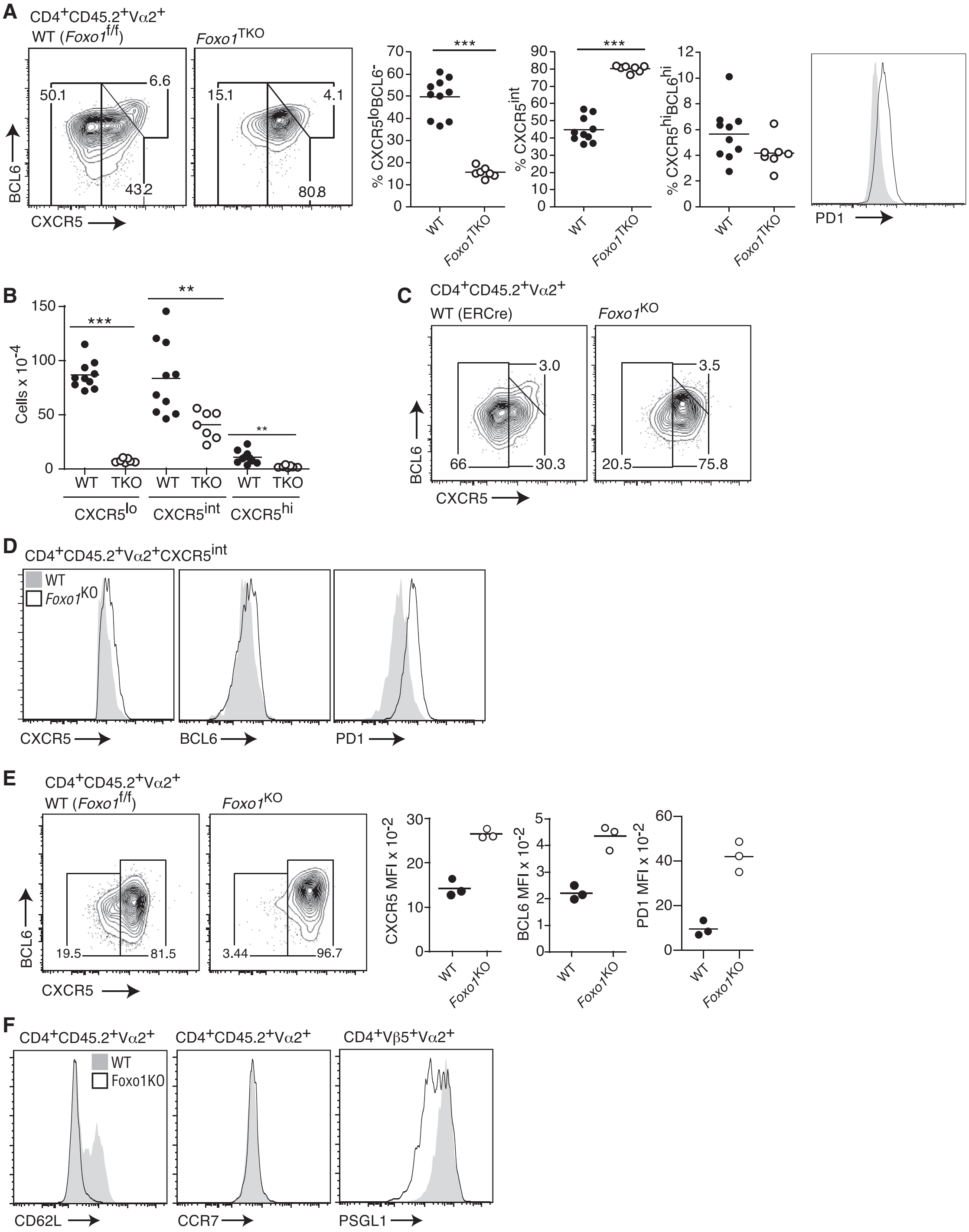

Figure 1. Loss of FOXO1 Amplifies Tfh Differentiation

(A and B) WT or *Foxo1*^{TKO} OTII cells were transferred into CD45.1 hosts, immunized with OVA plus adjuvant, and spleen cells were analyzed days 4 post immunization. (A) The percentages of CXCR5^{lo}, CXCR5^{int}, and CXCR5^{hi}BCL6^{+} cells (left) or PD1 expression (right) were determined by flow cytometry and (B) The numbers of each cell type in the spleen were calculated, representative of two independent experiments.

(C) Adoptive transfer similar to (A) above were carried out with *Foxo1*^{KO} OTII cells and the percentages of WT or *Foxo1*^{KO} CXCR5^{lo}, CXCR5^{int}, and CXCR5^{hi}BCL6^{+} cells were determined days 4 post immunization.

(D) Expression of CXCR5, BCL6, and PD1 from WT (filled histogram) or *Foxo1*^{KO} (open histogram) CXCR5^{int} OTII cells days 4 post immunization (n = 3). Representative of two independent experiments.

(legend continued on next page)

noted that these mice accumulate a large population of Tfh cells, form GCs, and produce circulating, anti-DNA antibodies, and we proposed that the PI3K-AKT-FOXO1 signaling pathway controls lineage commitment that, in part, specifies the Treg versus Tfh alternative cell fates (Kerdiles et al., 2010; Hedrick et al., 2012). Though provocative, these experiments highlight a necessity to study the role of FOXO transcription factors in T cell differentiation without the complications of autoimmunity caused by an insufficiency of Treg cells. In support of this idea, a report recently appeared showing that the ubiquitin ligase, ITCH, facilitates Tfh differentiation, and indeed it appears to act through the degradation of FOXO1 (Xiao et al., 2014). Here, we test the proposition that ICOS signaling acts to initiate a program of Tfh differentiation through inhibition of FOXO1 and the resulting effects on gene expression. Specifically, the deletion of *Foxo1* results in enhanced BCL6 expression and exaggerated differentiation of Tfh cells.

RESULTS

Loss of FOXO1 Amplifies Tfh Differentiation

In accord with the high prevalence of Tfh cells in mice with a T cell-specific *Foxo1* deletion (Kerdiles et al., 2010), we tested whether ICOS-mediated FOXO1 inactivation constitutes an important step in Tfh cell differentiation. As such, we adoptively transferred *Foxo1*$^{f/f}$*Cd4Cre*$^{+}$CD45.2^{+} (*Foxo1*^{TKO}) OTII or *Foxo1*$^{f/f}$CD45.2^{+} (wild type, WT) OTII cells into CD45.1 mice. In this and subsequent experiments, the starting population was depleted of CD25^{+}CD69^{+} cells prior to transfer. Host mice were then immunized with OVA plus adjuvant. Four days post-immunization WT and *Foxo1*^{TKO} OTII cells were fully activated as determined by CD44 expression (data not shown), and the WT OTII cells differentiated into three cell populations: CXCR5^{lo}BCL6^{lo} cells, described as T effector (Teff) cells; CXCR5^{int} cells, Tfh cells; and CXCR5^{hi}BCL6^{hi} T cells that are destined to be GC-Tfh cells (Pepper et al., 2011; Liu et al., 2013). By contrast, almost all *Foxo1*^{TKO} OTII cells displayed CXCR5^{int} expression characteristic of Tfh cells (Figure 1A). Consistent with this, PD1 expression was also elevated in *Foxo1*^{TKO} compared to WT T cells (Figure 1A).

Contrary to expectations given the role of FOXO transcription factors in the expression of Bim and Fas-ligand (Calnan and Brunet, 2008; Fu and Tindall, 2008), there was a decrease in the total number of *Foxo1*^{TKO} T cells compared with WT (Figure S1A). The analysis of cultured T cells showed that this defect in accumulation was not due to retarded cell division, but rather, increased apoptosis (Figure S1B–1F). It is cell-intrinsic (Figure S1D), and could be completely rescued by the addition of a pan-caspase inhibitor (Figure S1F). Although activation via interleukin-2 (IL-2) or a superantigen leads to FOXO1 inactivation (Stahl et al., 2002; Fabre et al., 2005), an important point is that this inactivation was transient, such that at least by 24 hr post-activation, FOXO1 contributed to CD4^{+} T cell survival.

All three populations were reduced with a *Foxo1* deletion, although the decrease was minimal for Tfh (CXCR5^{int}) cells (Figure 1B). IL-7 is required for naive T cell survival and normal expression of BCL2 in naive T cells, and it increases Tfh cell differentiation (Surh and Sprent, 2008; Seo et al., 2014). As Foxo1-deficient naive cells have reduced expression of IL-7Rα (Kerdiles et al., 2009), we determined whether enforced expression of *Il7ra* (Yu et al., 2004) would rescue survival or alter the course of the response. Results showed no effect of *Il7ra* expression on the proportion or number of *Foxo1*^{TKO} cells that became Tfh cells (Kerdiles et al., 2010; data not shown).

A *Foxo1* loss of function was further tested by acute deletion just prior to immunization. After treatment with tamoxifen, T cells were harvested from *Foxo1*$^{f/f}$ *Rosa26*$^{Cre\text{-}ERT2}$ OTII (*Foxo1*^{KO}) and *Rosa26*$^{Cre\text{-}ERT2}$ OTII mice (WT) (Kerdiles et al., 2009) and transferred into naive hosts. The starting and unimmunized OTII populations from these mice were equivalent for the expression of CD44 and CXCR5 (Figure S1G and data not shown). Notably, the proportion of *Foxo1*^{KO} OTII cells that acquired a CD44^{hi} activated phenotype day 4 post immunization was equivalent to WT, and yet similar to *Foxo1*^{TKO} T cells, nearly all *Foxo1*^{KO} OTII cells displayed a CXCR5^{int} phenotype (Figures 1C and S1G). Similar to *Foxo1*^{TKO} T cells, further analysis of this CXCR5^{int} Tfh subset revealed higher expression of CXCR5, BCL6, and PD1 in *Foxo1*^{KO} cells compared with the equivalent WT CXCR5^{int} population (Figures 1D and S1H).

To determine whether these effects applied to other immunization conditions, we analyzed the response to infection with *Listeria monocytogenes*. After adoptive transfer of OTII cells, host mice were infected with *actA*-deficient *Listeria monocytogenes* (ΔActA-Lm) expressing OVA (Ertelt et al., 2009), and the analysis day 4 post infection revealed that virtually all the *Foxo1*^{KO} OTII cells were CXCR5^{+} (Figure 1E). Again, within the CXCR5^{+} population, *Foxo1*^{KO} T cells were uniformly higher by approximately two-fold for the expression of CXCR5, BCL6, and PD1 (Figure 1E).

A defining characteristic of Tfh cells is location within the B cell follicles, whereas the eponymous GC-Tfh cells are located within GCs. To analyze the role of FOXO1 in localization, we determined the expression of homing molecules in addition to CXCR5. As expected, based on the control of *Klf2* by FOXO1 (Fabre et al., 2008; Kerdiles et al., 2009), virtually all *Foxo1*^{KO} OTII cells were CD62L^{-} 4 days post immunization, whereas the WT T cells displayed heterogeneous expression (Figure 1F). CCR7 expression was unchanged with respect to activated WT T cells, but a proportion of the *Foxo1*^{KO} OTII cells were low for PSGL1 (Figure 1F), a phenotype that allows T cells to exit the T cell zone (Crotty, 2014). Combined with the expression of CXCR5 (e.g., Figure 1D), *Foxo1*^{KO} OTII cells appear to express a repertoire of homing molecules that would promote homing to B cell areas of the spleen (Crotty, 2014).

WT or *Foxo1*^{KO} OTII T cells were directly examined 4 days after immunization by immunohistology. WT OTII cells were mostly found within the splenic T cell zone including some cells along

(E) Analysis of CXCR5 versus BCL6 expression of WT *Foxo1*^{KO} cells 4 days post infection with ΔActA-Lm expressing OVA (Left). Plots show CXCR5, BCL6, or PD1 MFI (Right). $p < 0.01$ for all three parameters. Data is representative of two individual experiments.

(F) Expression of CCR7, CD62L, and PSGL1 on WT or *Foxo1*^{KO} OTII cells days 4 post immunization is shown (n = 3–4). Data are representative from at least two individual experiments.

the T cell-B cell border. In contrast, a larger proportion of the *Foxo1*^{KO} OTII cells was found in the follicle with relatively few cells found deep within the T cell zone (Figure S1I). However, we note that the *Foxo1*^{KO} cells were also not found deep in the B cell follicle.

The Regulation FOXO1 and ICOS Is Coupled via a Negative Feedback Loop

To analyze the relationship between ICOS signaling and FOXO1, we tested whether ICOS signaling would inactivate FOXO1 via nuclear egress (Calnan and Brunet, 2008). Naive CD4^{+} T cells expressing a FOXO1-GFP fusion protein were activated for 48 hr under iTfh conditions, rested for 24 hr, and restimulated for 30 min with antibody specific for CD3 in the presence or absence of agonist ICOS-specific antibody. At 30 min post restimulation there was no difference in the amount of FOXO1-GFP in live cells (Figure 2A, left). However, upon restimulation through CD3 and ICOS, but not CD3 alone, the similarity score (ImageStream analysis) for DRAQ5 (nucleus) and FOXO1-GFP was reduced; this corresponds with reduced co-localization and nuclear FOXO1 (Figure 2A, middle). In agreement, there was an increased percentage of cells stimulated through ICOS that displayed FOXO1-GFP exclusively in the cytoplasm (Figure 2A, right). However, at 24 hr post restimulation through CD3 and ICOS, the amount of FOXO1-GFP was increased with little difference in the DRAQ5, FOXO1-GFP similarity score (Figure 2B, left, middle). Consistent with these results, nuclear intensity of FOXO1-GFP was not diminished in live cells 24 hr post-restimulation through CD3 and ICOS (Figure 2B, right). These observations show nuclear FOXO1, which was lost at 30 mim post-activation, was reestablished by 24 hr.

FOXO transcription factors have been shown to positively regulate the transcription of growth factor receptors (e. g., IL-7Rα, insulin receptor) that, in turn, signal through PI3K to cause FOXO inactivation (Hedrick, 2009; Kerdiles et al., 2009). This creates a negative feedback loop. Activation through CD3 and CD28 induced ICOS expression in WT T cells, and this induction was attenuated in *Foxo1*^{KO} T cells (Figure 2C, left). Because ICOS signaling also inactivated FOXO1, how is ICOS maintained in differentiating Tfh cells? To examine this, we further measured ICOS expression in iTfh cultures and found that ICOS was super-induced in WT T cells consistent with the phenotype of Tfh cells, and its expression became relatively less FOXO1 dependent (Figure 2C, right). *Foxo1*^{KO} T cells cultured in iTfh conditions expressed an amount of ICOS at least equivalent to WT T cells co-stimulated through CD28. A conclusion is that although ICOS could be potentially subject to negative feedback regulation, there are two ways in which this is tempered. One, ICOS-mediated FOXO1 inactivation is transient (Figures 2A and 2B), and two, FOXO1 dependence is reduced under iTfh conditions (Figure 2C, right). In vivo activation also revealed ICOS induction compared with naive T cells, and its expression was progressively higher comparing Teff (CXCR5^{lo}), Tfh cells (CXCR5^{int}) and GC-Tfh (CXCR5^{hi}BCL6^{hi}) cells. In all three subsets, the ICOS induction was partially dependent upon FOXO1 (Figure 2D).

The results suggested the possibility that FOXO1 directly regulates *Icos* expression. To analyze FOXO1 chromosomal binding in naive T cells, we carried out a whole-genome scan for FOXO1 binding sites in CD4 T cells (ChIP-seq) (Hess Michelini et al., 2013). Accuracy of the analysis was verified by an examination of the average tags per position, genomic GC content, and the distribution of peaks between regions of the genome (Figure 2E). The most frequent binding site corresponded with the known FOXO-DAF16 consensus site (Figure 2E) (Hedrick et al., 2012). In addition, the analysis pinpointed binding sites in the *Il7r* and *Ctla4* genes we have previously identified as evolutionarily conserved and bound by FOXO1 (Kerdiles et al., 2009; Kerdiles et al., 2010) (Figure S2A). These data further revealed that in CD4 T cells, FOXO1 is bound to an evolutionarily conserved FOXO consensus binding site in the *Icos* promoter (Figures 2F and S2B) and remains bound after activation for 48 hr (Figure 2G). Thus, similar to *Il7ra* and *Ctla4*, *Icos* expression is dependent in part on FOXO1, and the *Icos* gene is bound by FOXO1 at an evolutionarily conserved promotor binding site.

Tfh Cell Differentiation in the Absence of FOXO1 Is Independent of ICOSL

FOXO1-deficient T cells have diminished expression of ICOS, and yet exhibit enhanced Tfh differentiation. This, combined with the ICOS-dependent inactivation of FOXO1 suggested that genetic ablation of FOXO1 would promote ICOS-independent Tfh differentiation. To test this, we analyzed the dependence of Tfh differentiation on ICOSL in two ways. In one set of experiments, we transferred WT or *Foxo1*^{KO} T cells in the presence or absence of antibodies specific for ICOSL. In a second set of experiments, we transferred T cells into WT or *ICOSL*$^{-/-}$ hosts. In these experiments, the results were similar. Although the presence of WT Tfh cells displayed a strong dependence on ICOSL recognition, this dependence was greatly reduced for *Foxo1*^{KO} T cells (Figure 3A–3D). Importantly, in both experimental models, the number of *Foxo1*^{KO} CXCR5^{+} OTII cells was substantially greater than the number of WT CXCR5^{+} cells under these conditions (Figures 3B and 3D). In particular, although the differentiation of WT cells was virtually lost in *Icosl*$^{-/-}$ hosts (Figures 3C and 3D) (Choi et al., 2011; Pepper et al., 2011), in the absence of FOXO1 the mean *number* of CXCR5^{+} T cells was increased by 10-fold over WT controls (Figure 3D). Further experiments showed that CXCR4 induction, shown to have a stringent requirement for ICOS in WT T cells (Odegard et al., 2008) was induced in *Foxo1*^{KO} T cells in an ICOS-independent manner (Figures S3A and S3B). From these data, we conclude that loss of FOXO1 facilitates differentiation into Tfh cells with a greatly diminished requirement for ICOS signaling, i.e. FOXO1 inactivation is epistatic to ICOS expression and signaling.

Loss of FOXO1 Promotes B Cell Help and Anti-DNA Antibodies in the Absence of ICOS

To determine whether loss of *Foxo1* could complement a loss of *Icos*, we bred *Foxo1*^{TKO} with *Icos*$^{-/-}$ mice and analyzed the proportion of CXCR5^{+}PD1^{+} cells from each of four genotypes. To account for the increase in activated CD4 cells in the *Foxo1*^{TKO} mice and the reduced population of activated cells in *Icos*$^{-/-}$ mice (Odegard et al., 2008; Kerdiles et al., 2010), we focused on the activated CD4^{+} (CD44^{hi}) population. In addition, we enumerated class-switched and GC B cells. For each of these parameters, the deficiencies displayed by *Icos*$^{-/-}$ mice were all or partially rescued by the inclusion of the *Foxo1*^{TKO} alleles

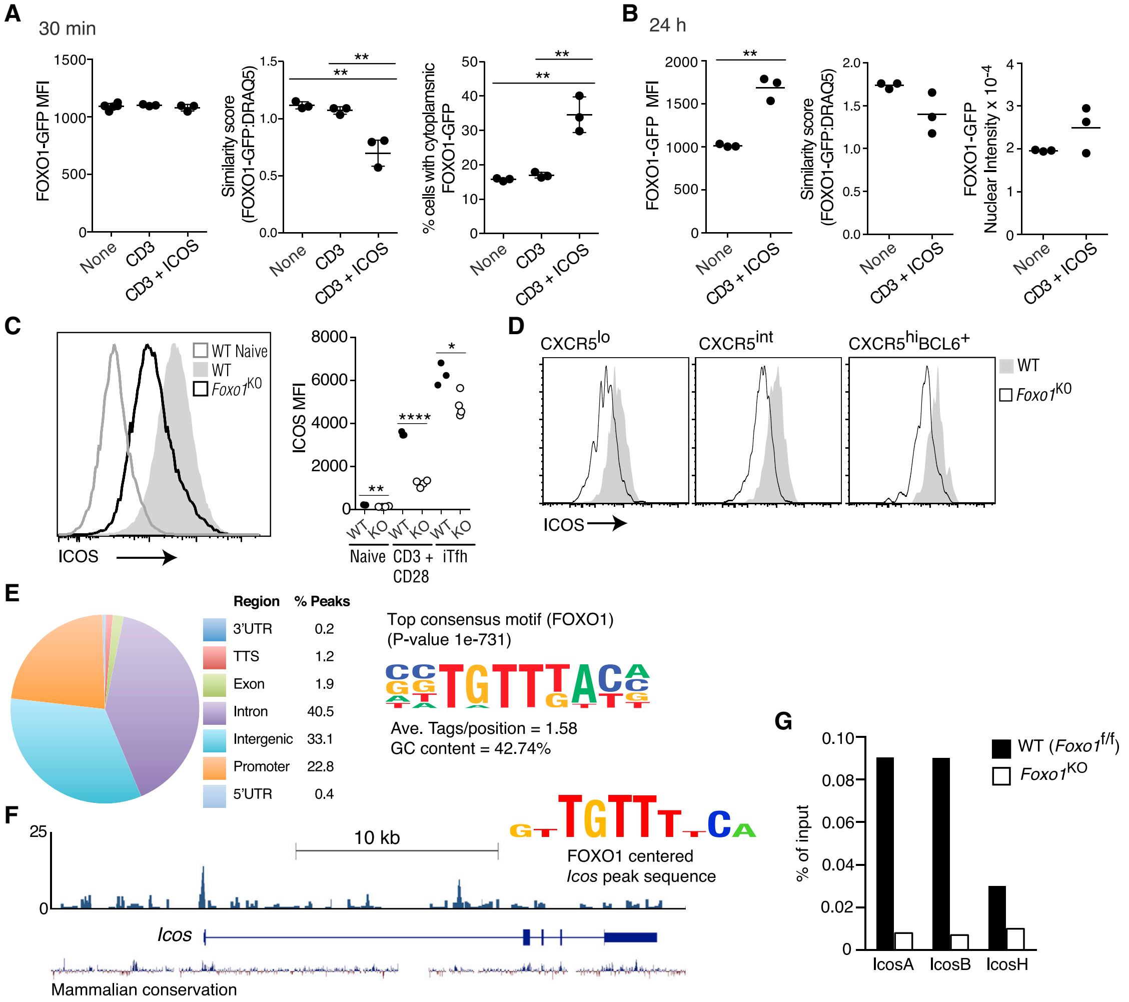

Figure 2. The Regulation FOXO1 and ICOS Is Coupled via a Negative Feedback Loop

(A) Plots show MFI of FOXO1-GFP (left), similarity score between DRAQ5 and FOXO1-GFP (Middle), and the percent of cells with FOXO1-GFP exclusively in the cytoplasm (right) 30 min post restimulation through CD3 and ICOS. None indicates the cells were not restimulated. Data are representative of two independent experiments.

(B) Plots show MFI of total FOXO1-GFP (left), similarity score between DRAQ5 and FOXO1-GFP (middle), and intensity of FOXO1-GFP overlapping with the nuclear mask (right) 24 hr post restimulation.

(C) ICOS expression on WT or *Foxo1*^{KO} CD4 cells activated in vitro for 72 hr via CD3 and CD28 in the presence or absence of iTfh conditions. The histograms show the expression of ICOS on cells activated with anti-CD3 and anti-CD28 without the addition of exogenous cytokines (left). Data are representative of two independent experiments.

(D) Histograms depict ICOS expression on CXCR5^{lo}, CXCR5^{int}, or CXCR5^{hi}BCL6^{+} from WT or *Foxo1*^{KO} OTII cells days 4 post immunization. Data are representative of two independent experiments.

(E) Analysis of FOXO1-specific ChIP-Seq of naive CD4 T cells. The most frequent consensus binding site was determined to be TGTTTAC, the size of the nucleotide in the graphic corresponds with its frequency.

(F) The *Icos* locus is shown for FOXO1-specific ChIP-seq (top track) (see also Figure S2B), and the centrally positioned nucleotide sequence within the promoter peak is listed. The bottom track shows mammalian sequence conservation (UCSC Genome Browser).

(G) FOXO1-specific ChIP of *Icos* locus from WT CD4 T cells activated in vitro.

(Figures 4A–4C). Although little to no immunoglobulin G (IgG) isotype anti-DNA antibodies were detected in the *Icos*$^{-/-}$ mice, significant titers were measured in DKO mice (Figure 4D, left). DKO mice also had significantly higher levels of total IgG levels in the sera than *Icos*$^{-/-}$ mice (Figure 4D, right). The presence of GCs and isotype switched antibodies was not simply due to a lack of regulatory FOXP3^{+} Tfh (T$_{FR}$) cells, beacuse the frequency of the CXCR5^{+} Tfr population within the Treg population was not reduced with the deletion of *Foxo1* (Figure 4E). These data indicate that deletion of *Foxo1* in T cells is sufficient to allow differentiation of a Tfh-like cell in the absence of ICOS, and these cells cooperate with B cells to produce isotype-switched, anti-DNA antibodies—at least in the absence of effective Treg cells.

FOXO1 Negatively Regulates BCL6 Expression

If the loss of FOXO1 is important for Tfh differentiation, then a prediction is that FOXO1 inhibition as a consequence of ICOS signaling will facilitate the induction of BCL6 expression (Choi

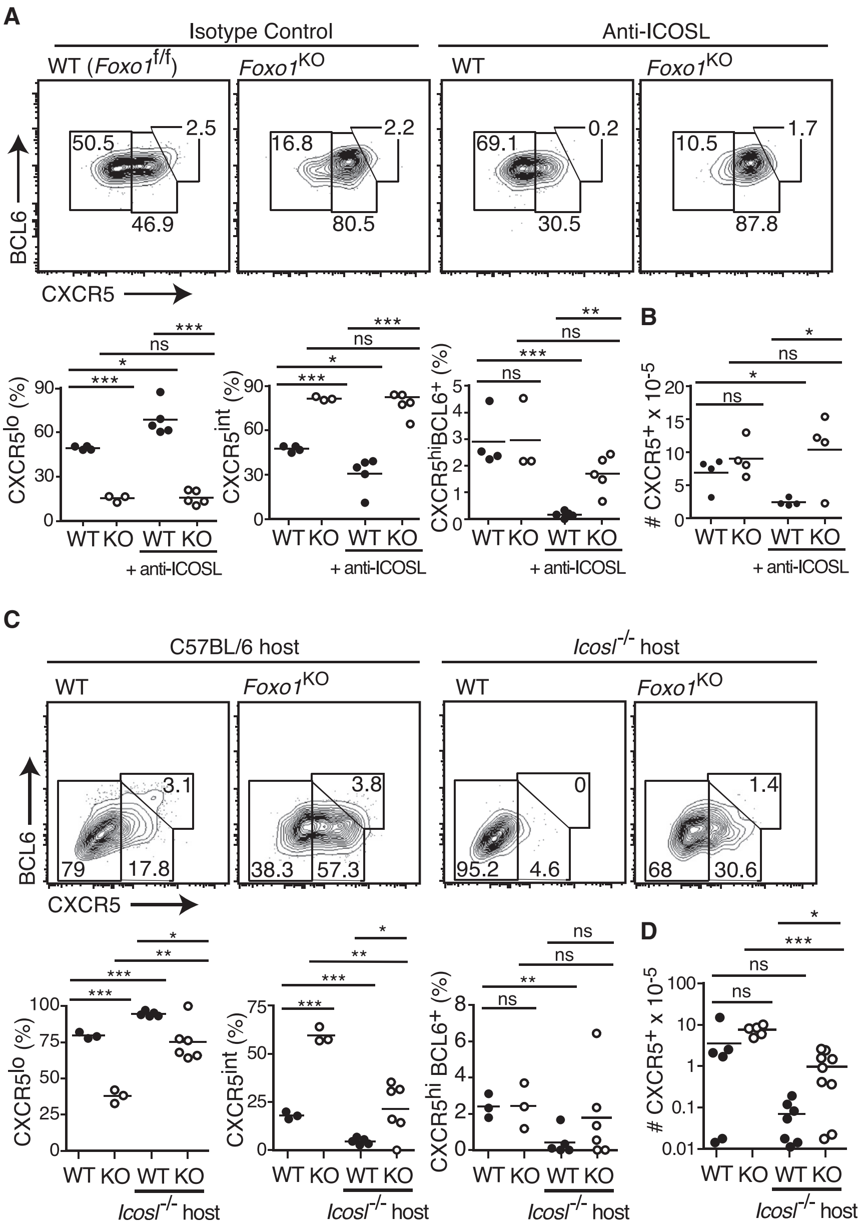

Figure 3. Tfh Differentiation in the Absence of FOXO1 Is Independent of ICOSL

(A and B) WT or *Foxo1*^{KO} OTII cells were transferred into CD45.1 hosts and mice were immunized with OVA plus adjuvant. Where indicated, mice were treated with blocking anti-ICOSL. (A) The percentages of CXCR5^{lo}, CXCR5^{int}, and CXCR5^{hi}BCL6^{+} cells and (B) total number of CXCR5^{+} (including both the CXCR5^{int} and CXCR5^{hi} populations) of WT or *Foxo1*^{KO} OTII cells is shown. One of four representative experiments.

(C) The percentages of CXCR5^{lo}, CXCR5^{int}, and CXCR5^{hi}BCL6^{+} cells of WT or *Foxo1*^{KO} OTII cells in WT or *Icosl*$^{-/-}$ hosts days 4 post immunization. Data are representative of two independent experiments.

(D) Numbers of WT or *Foxo1*^{KO} CXCR5^{+} (including both the CXCR5^{int} and CXCR5^{hi} populations) OTII cells days 4 post immunization from WT or *Icosl*$^{-/-}$ hosts plotted on a log scale. Data are pooled from two independent experiments.

et al., 2011). In naive cells, the low amount of BCL6 detected was unchanged between WT and *Foxo1*^{KO} mice (data not shown). T cells were activated for 48 hr under iTfh conditions, and they were rested for 24 hr and re-stimulated with or without ICOS-specific antibody for a further 24 hr. Restimulation through ICOS increased BCL6 expression, whereas it was substantially higher in *Foxo1*^{KO} T cells compared with WT T cells under all conditions (Figure 5A). In particular, *Foxo1*^{KO} T cells re-stimulated through CD3 alone expressed more BCL6 than WT T cells stimulated through CD3 and ICOS. There was a further induction of BCL6 in the *Foxo1*^{KO} T cells stimulated through ICOS (compare anti-CD3 with anti-CD3 plus anti-ICOS), and this suggests that an additional pathway downstream of ICOS might play a role in BCL6 induction. Similar to protein expression, WT *Bcl6* RNA increased upon restimulation in the presence of anti-ICOS, and it was expressed in higher amounts in *Foxo1*^{KO} cells compared to WT cells (Figure 5B). Furthermore, this increase in BCL6 was cell-intrinsic (Figure 5C), and it was not secondary to selective death of *Foxo1*^{KO} T cells (Figure S4A).

Analysis of FOXO1 binding by ChIP-Seq in naive CD4 T cells showed that FOXO1 is exclusively bound to the *Bcl6* locus at the boundary of the first (38 bp) non-coding exon and the first intron (Figures 5D and S4B). This region includes tandem sequences separated by 30 bases that are very similar to the conserved FOXO1 consensus site (Figures 5D and S4B), and we have also found this peak in naive and activated CD8 T cell data sets (data not shown). This region is highly conserved between mice and human beings and this conservation extends to a comparison of marsupials and eutherian mammals, implying evolutionary selection for at least 130 million years (Figure S4C).

FOXO1 binding to this site in naive T cells was confirmed by ChIP analysis (Figure 5E). We further examined whether FOXO1 binding is lost under conditions of T cell stimulation. After 48 hr of iTfh activation, cells were rested for 24 hr and tested (None), or re-stimulated through CD3 and ICOS for 1 hr or 24 hr. As shown, FOXO1 was bound to this site in T cells activated under iTfh conditions, but it was reduced upon restimulation with through CD3 and ICOS (Figure 5F). This is consistent with the initially reduced nuclear localization of FOXO1 (Figure 2A). However, nuclear FOXO1 is not decreased 24 hr post-restimulation through CD3 and ICOS (Figure 2B), and yet binding of FOXO1 to *Bcl6* was still reduced (Figure 5F). These data are consistent with FOXO1 binding to the *Bcl6* gene and mediating transcriptional repression that is relieved upon ICOS signaling; however, we lack direct evidence for transcription repression

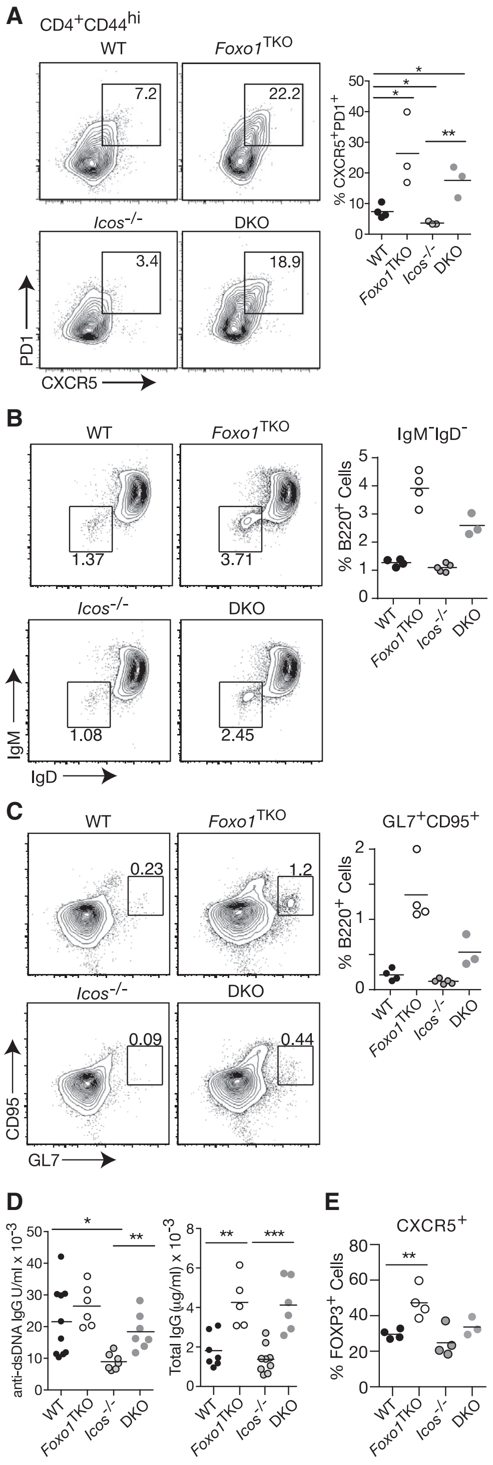

Figure 4. Loss of FOXO1 Promotes B Cell Help and Antibodies in the Absence of ICOS

(A) The percentage of LN $CD4^+CD44^{hi}$ cells expressing CXCR5 and PD1 is shown from WT; *Foxo1*^{TKO}; *Icos*$^{-/-}$; and DKO. Data are representative of four independent experiments.

(B and C) The percentages of isotype switched (IgM^-IgD^-) (B) and GC ($GL7^+CD95^+$) (C) B cells present are shown. Comparing WT and *Foxo1*^{TKO} or *Icos*$^{-/-}$ and DKO $p < 0.01$. Data are representative of four independent experiments.

(D) Plot shows relative amounts of IgG anti-dsDNA in sera. Data are representative of three independent experiments (left). The levels of total IgG in sera are plotted. Data shown are pooled from two experiments (right).

(E) Plots show the percentage of $CXCR5^+$ T_{FR} cells within LN Treg ($CD4^+$ $FOXP3^+$) population for each genotype. Data shown are from one of two independent experiments.

that might include germline mutations in the tandem FOXO1 binding sites.

Further analysis of the 4333 FOXO1 genomic binding sites revealed many genes involved in Tfh differentiation located within close proximity. Inspection of *Cxcr5*, *Batf*, *Ccr7*, *Cxcr4*, *Irf4*, *Selplg* (P-selectin ligand-CD162), and *Maf* loci revealed one or more strong FOXO1 binding sites located near the transcriptional start site or within several kilobases (Figure S5). The exception was *Maf*, which is functionally important for terminally differentiated GC-Tfh cells (Liu et al., 2013).

Enforced Nuclear Localization of FOXO1 Prevents Tfh Differentiation

If FOXO1 inactivation is required for Tfh differentiation, enforced nuclear localized would be predicted to block the appearance of Tfh cells. To test this, we transduced T cells from OTII *Foxo1*^{AAA} mice with a Hit and Run CRE recombinase retrovirus and adoptively transferred them (Silver and Livingston, 2001; Ouyang et al., 2012). After immunization, *Foxo1*^{AAA} T cells expressed $CD44^+$ (data not shown) and displayed superinduction of ICOS (Figure 6A) consistent with the importance of FOXO1 in the regulation of the *Icos* gene. Despite this, *Foxo1*^{AAA} T cells displayed a reduced ability to differentiate into the Tfh phenotype as compared to WT (Figure 6B). The accumulation of *Foxo1*^{AAA} T cells was also reduced (data not shown), and the origin of this defect is a topic of further investigation.

Foxo1^{KO} T Cells Have Reduced Ability to Differentiate into GC-Tfh Cells

To characterize the role of FOXO1 in GC-Tfh differentiation, we examined a polyclonal response to *L. monocytogenes*. For this, we generated mixed WT:*Foxo1*^{TKO} bone-marrow chimeras (Kerdiles et al., 2010). Mice were infected with ΔActA-Lm, and at day 9 the $CXCR5^{int}$ (and total $CXCR5^+$ cells) cells were overrepresented within the *Foxo1*^{TKO} population compared with WT cells. Surprisingly there was a notable paucity of *Foxo1*^{TKO} $CXCR5^{hi}BCL6^{hi}$ GC-Tfh cells (Figure 7A).

Studies have shown that MAF is an important transcription factor in Tfh development, and in particular, it might be essential for IL-4 expression associated with GC-Tfh cells (Liu et al., 2013; Crotty, 2014; Ueno et al., 2015). Consistent with this, analysis of WT OTII T cells 4 days after activation in vivo revealed that only the $CXCR5^{hi}$ $PD1^{hi}$ subset expressed high amounts of MAF (Figure 7B), and this was abrogated by treatment of the mice with anti-ICOSL (Figure 7C). Compatible with the lack of a $BCL6^{hi}$ population at GC time points, *Foxo1*^{KO} T cells were selectively deficient in the MAF^+ population at both day 4 and day 7 post immunization (Figure 7D). In addition, *Foxo1*^{KO} OTII T cells did not give rise to $CXCR5^{hi}BCL6^{hi}$ or $CXCR5^{hi}PD1^{hi}$ cells day 7 post immunization (Figure 7E), and at this time, FOXO1 is consistently expressed (Figure 7F). Similar results were found following infection with VSV-OVA (Figures 7G and 7H). In sum, these data show

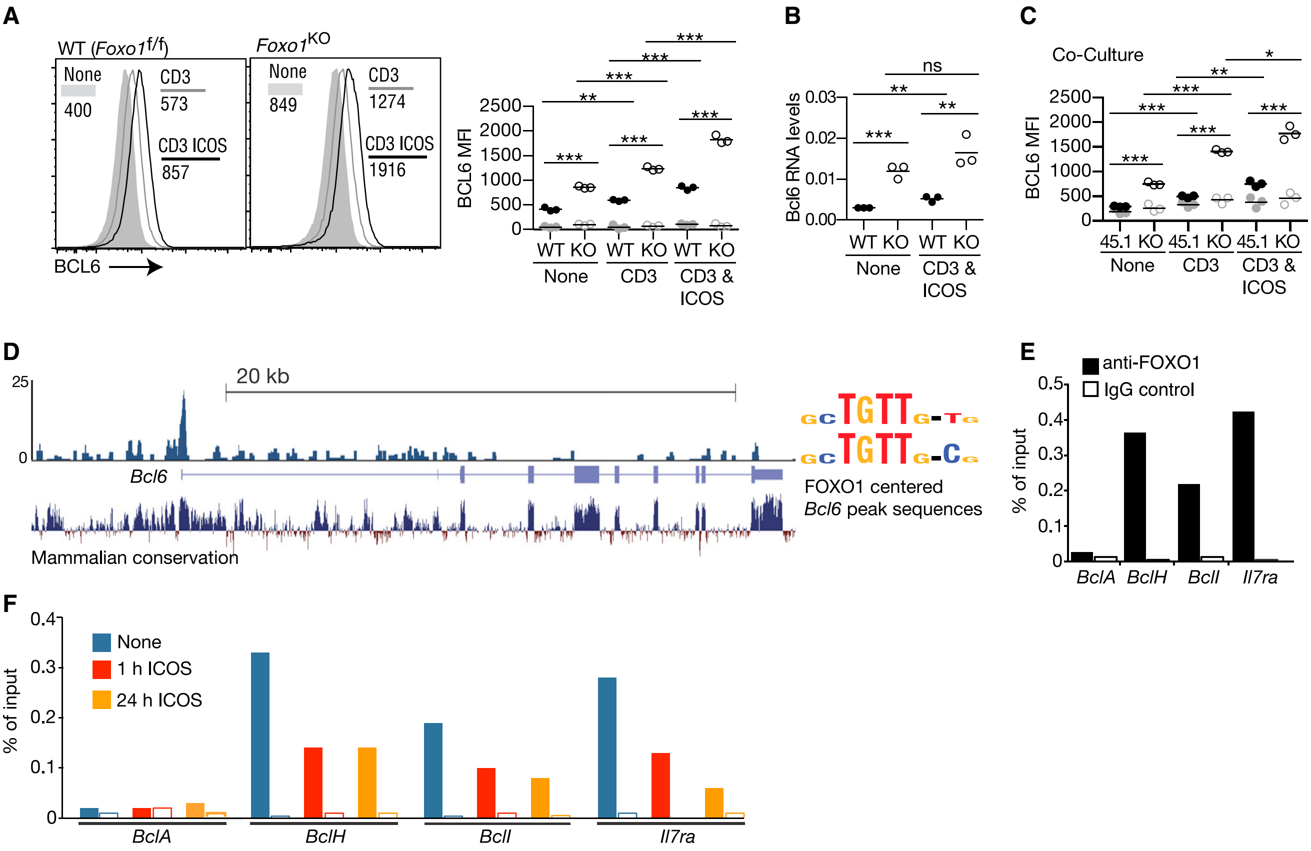

Figure 5. FOXO1 Negatively Regulates BCL6 Expression

(A and B) WT or *Foxo1*^{KO} naive CD4 cells activated under iTfh conditions, rested, and then restimulated via CD3 with or without antibody specific for ICOS. (A) BCL6 levels determined by flow cytometry. None indicates the cells were not restimulated, and the MFI is shown for one example. The filled (WT) and open (*Foxo1*^{KO}) gray circles on the graph represent background staining of a control antibody for each individual biological replicate. Data are representative of three independent experiments. (B) *Bcl6* levels were determine by qPCR. Data shown are from one of two experiments.

(C) WT (CD45.1) and *Foxo1*^{KO} (CD45.2) cells were co-cultured under conditions as in (A) and the MFI of WT and *Foxo1*^{KO} cells from each well are shown. Gray circles represent background staining of a control antibody. Data are representative of two individual experiments.

(D) The *Bcl6* locus is shown for FOXO1-specific ChIP-Seq (top track) (see also Figure S4B), and the centrally positioned nucleotide sequences within the peak found in the first intron are listed. The bottom track represents mammalian sequence conservation.

(E and F) FOXO1-specific ChIP of *Bcl6* locus from (E) naive CD4 T cells or (F) CD4 T cells activated as in (A) and restimulated with CD3- and ICOS-specific antibodies as in (A) for 1 hr or 24 hr. Filled bars represent percent of input of anti-FOXO1 immunoprecipitation. Open bars represent percent of input of the IgG control. None indicates the cells were activated and rested but not restimulated. Data are representative of two independent experiments.

that genetic inactivation of *Foxo1* exaggerates the differentiation of Tfh cells in an ICOS-independent manner, and yet, FOXO1 plays a role in the final differentiation to GC-Tfh cells.

DISCUSSION

Previous work established an early role for ICOS and its activation of PI3K signaling in the differentiation of CD4 T cells into Tfh cells, and this signaling pathway influences the induction of key molecules including BCL6, MAF, IL-4, and IL-21 (Bauquet et al., 2009; Gigoux et al., 2009; Rolf et al., 2010a; Choi et al., 2011). Since the basis for PI3K regulation of cell growth and differentiation largely emanates through AKT-mediated inhibition of FOXO1 transcriptional activity (Calnan and Brunet, 2008), we wished to test the idea that *Foxo1* is epistatic to *Icos* in the elaboration of one or more of these Tfh characteristics. Additionally, two recent papers suggest that reduced expression of FOXO1, either due to increased expression of ICOS induced by loss of FOXP1, or due to ITCH-mediated degradation, may increase Tfh differentiation (Wang et al., 2014; Xiao et al., 2014). The studies described in this report provide a mechanism for those findings.

Tfh cells at the B-follicular border express CXCR5 and BCL6 (Ramiscal and Vinuesa, 2013), whereas GC-Tfh cells can be characterized by MAF expression. Here we show that deletion of *Foxo1* exaggerated the initial antigen-driven step in Tfh differentiation resulting in an expanded proportion of CXCR5^{+} CD4 T cells localized to the border of B cell follicles. *Foxo1*^{KO} T cells were proportionately overrepresented as CXCR5^{int} BCL6^{int} cells, and in addition these Tfh cells expressed amounts of CXCR5, BCL6, PD1, and CXCR4 greater than those of the equivalent WT Tfh populations—although not to the level characteristic of GC-Tfh cells. In fact, in the absence of FOXO1, despite the increased proportion of Tfh cells, few GC-Tfh cells emerged

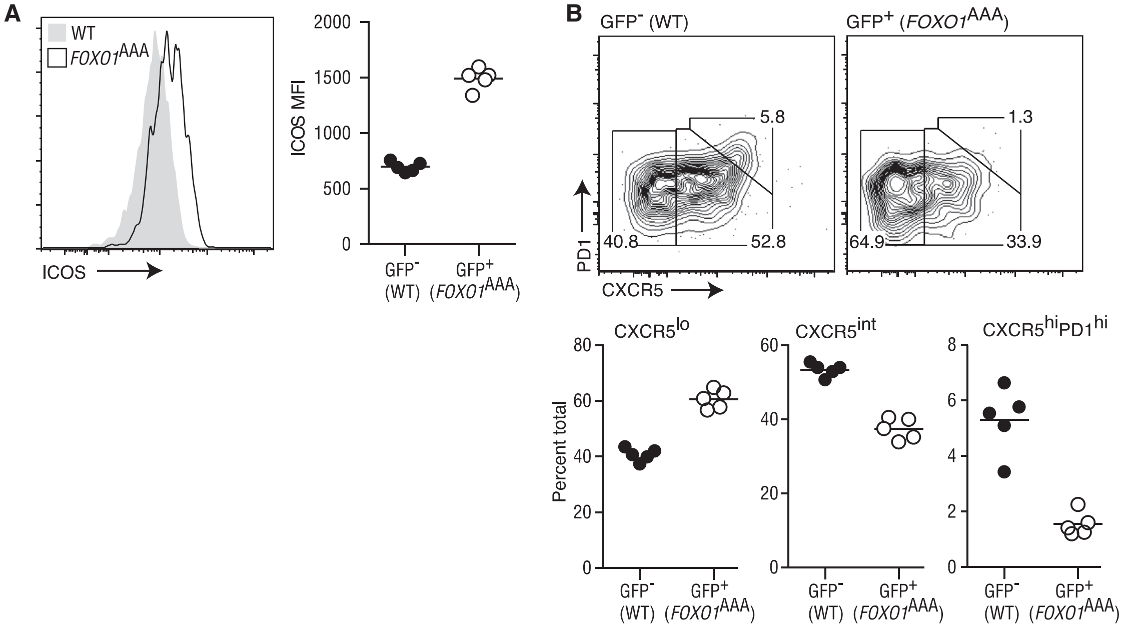

Figure 6. Enforced Nuclear Localization of FOXO1 Suppresses Tfh Differentiation

(A) ICOS expression or (B) the percentages of CXCR5^{lo}, CXCR5^{int}, and CXCR5^{hi}PD1^{+} cells from WT or *Foxo1*^{AAA} OTII cells day 4 post immunization. In each case, $p < 0.001$. Data are representative of two independent experiments.

even as late as 9 day post ΔActA-Lm infection. Our conclusion is that a transient inactivation of FOXO1 skews the contingency of effector versus Tfh differentiation, whereas progression to mature GC-Tfh cells is promoted by FOXO1.

We emphasize that FOXO1 inactivation is only transient. In T cells stimulated through CD3 and ICOS, nuclear FOXO1-GFP is reduced at 30 min but reestablished within 24 hr. Moreover, FOXO1 is required for T cell viability as early as 24 hr post activation. Whether there are mechanisms opposing AKT signaling or desensitizing ICOS signaling is not known; however, stress kinase phosphorylations, glycosylation, or methylation have all been shown to encourage nuclear location of FOXO factors (Hedrick et al., 2012). This is further illustrated by regulation of ICOS. Although FOXO1 clearly has a role for full ICOS expression, ICOS is induced early in DC-mediated antigen presentation, and remains high in Tfh and GC-Tfh cells despite its potential for signaling via PI3Kδ and causing negative feedback inactivation of FOXO1. Thus, although genetic ablations presented here and elsewhere point to an important contingency-based inactivation of FOXO1 (Wang et al., 2014; Xiao et al., 2014), they do not recapitulate the dynamics of FOXO1 inactivation. Furthermore, FOXO1 appears to be required for GC-Tfh differentiation, although the mechanism of action is unknown. The reduced expression of ICOS might limit the ability of *Foxo1*^{KO} Tfh cells to generate filopodia, which allow for Tfh cells to home from the T-B border to the GC (Xu et al., 2013). This possibility would be consistent with the presence of GC-Tfh cells in *Foxo1*^{TKO} mice contrasted with the loss of *Foxo1*^{KO} GC-Tfh cells in competition with WT cells. FOXO1 has also been shown to bind to the *Ifng* locus and inhibit expression of IFN-γ (Ouyang et al., 2012), and thus in its absence, ectopic gene expression might subvert GC-Tfh differentiation. The most parsimonious explanation is that FOXO1 directly regulates the transcription of genes required for full GC-Tfh differentiation.

A complication described here is the observation that activated *Foxo1*$^{-/-}$ CD4 T cells have a reduced viability compared to WT T cells. This raised the possibility that the increase in the proportion of Tfh cells could be due to selective death of Teff cells; however, the results show that this alone cannot explain the phenotype of *Foxo1*$^{-/-}$ T cells. If the exaggerated proportion of Tfh cells were due only to preferential loss of Teff cells, then there would be no reduction in the requirement for ICOS signaling. In two different types of experiments we show that *Foxo1*^{KO} T cells differentiate into Tfh cells with a substantially reduced requirement for ICOS signaling. Similarly, the induced expression of BCL6 is an important part of the Tfh program, and loss of FOXO1 results in the increased expression of BCL6 compared to wild-type, even when apoptosis is blocked. In a separate line of experimentation, loss of *Foxo1* genetically complemented the loss of *Icos* in that there emerged CXCR5^{+}PD1^{+} cells, GC-B cells and anti-DNA IgG antibodies. In addition, a role for FOXO1 in Tfh differentiation is supported by the known signaling pathway downstream of ICOS in T cells, that is, PI3K and AKT activation (Rolf et al., 2010b), which was shown here to result in the inactivation of FOXO1. Finally, enforced nuclear expression of FOXO1 inhibits the differentiation Tfh cells, and the sum of these results provide a mechanism by which ITCH-mediated FOXO1 degradation is required for Tfh differentiation (Xiao et al., 2014).

These results demonstrate that inactivation of FOXO1 is an essential outcome of ICOS signaling in the contingency of CD4 T cell differentiation, and this establishes an important link in the signaling from ICOS to the induction of *Bcl6* expression. Previous studies have reported that FOXO1, FOXO3, or FOXO4

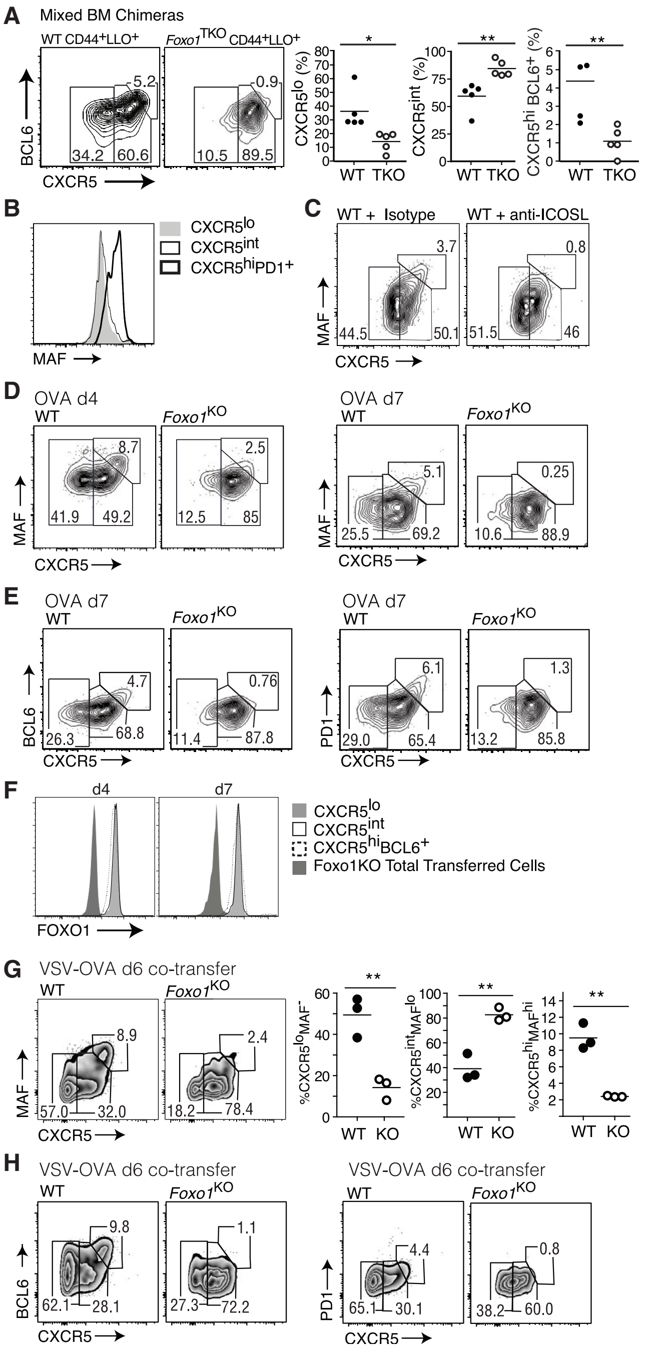

Figure 7. FOXO1 KO T Cells Have Reduced Ability to Differentiate into GC-Tfh Cells

(A) Mixed bone-marrow chimeras (WT-CD45.1 and *Foxo1*^{TKO}) were infected with ΔActA-Lm. CD4^{+}B220^{-}CD44^{hi}LLO^{+} cells were phenotyped from each donor. Representative of two separate experiments.

(B) MAF expression within CXCR5^{lo}, CXCR5^{int}, or CXCR5^{hi}PD1^{hi} OTII T cells day 4 post immunization (n = 3). Representative of three independent experiments.

(C) Percentages of CXCR5^{lo}, CXCR5^{int}, and CXCR5^{hi}MAF^{hi} subsets within CD4^{+} WT OTII T cells day 4 post immunization in the presence or absence of blocking anti-ICOSL. Representative of four host mice per condition.

(D) Analysis similar to (C) using WT or *Foxo1*^{KO} OTII cells assayed at day 4 (n = 3–5) and day 7 (n = 3). One of at least two representative experiments.

(E) Plots show CXCR5 versus BCL6 (left) or CXCR5 versus PD1 (right) at day 7 post immunization. One of two representative experiments.

(F) Expression of FOXO1 in CXCR5^{lo}, CXCR5^{int}, and CXCR5^{hi}BCL6^{+} subsets of WT OTII cells at day 4 and day 7 post immunization.

(G) The proportions of CXCR5^{lo}, CXCR5^{int}, or CXCR5^{hi}MAF^{+} cells at day 6 post VSV-OVA infection from co-transferred WT and *Foxo1*^{KO} OTII T cells. Data are representative of two independent experiments.

(H) Plots show CXCR5 versus BCL6 (left) or CXCR5 versus PD1 (right) expression from cotransferred WT or *Foxo1*^{KO} OTII T cells at day 6 post VSV-OVA infection (as in G). Data are representative of two independent experiments.

binds upstream of *BCL6* acting as a postive regulator in different types of cells (Pellicano and Holyoake, 2011; Oestreich et al., 2012), whereas we found that a *Foxo1* deletion enhances BCL6 expression. We also found, using ChIP-seq, that FOXO1 binding in naive CD4 T cells was restricted to a site at the beginning of the *Bcl6* first intron (also the case for naive and activated CD8 T cells—data not shown), and we propose that FOXO1 regulates *Bcl6* in T cells through transcriptional repression. Repression at this region is also associated with STAT5 competition for STAT3 binding (Walker et al., 2013). In addition, this region of the first *BCL6* intron is often mutated in diffuse large B cell lymphomas (DLBCL) (Migliazza et al., 1995). The mechanisms of *Bcl6* regulation in T cells are not as well studied, although there is evidence for contributions from STAT3, STAT5, and BATF (Liu et al., 2013).

Combined with previous results showing that FOXO1 is required for Treg differentiation (Kerdiles et al., 2010; Ouyang et al., 2010), a possibility is that the extent or duration of FOXO1 nuclear exclusion is one factor determining the fate of antigen-activated CD4 T cells. Whether the contingency decision is simply stochastic or depends upon an undetermined variable such as strength of signal (TCR peptide-MHC affinity or avidity), concentration of free cytokines, or location, is unknown. Nonetheless, the differential requirements for FOXO1 activity likely explain why Tfr cells derive from tTregs and not pTregs (Chung et al., 2011; Linterman et al., 2011). Naive T cells could not simultaneously receive an ICOS signal and maintain FOXO1 activity—both of which would be required for Tfr differentiation from naive T cells (Hedrick et al., 2012; Sage et al., 2013). Rather, tTregs differentiate into stable Tregs in the thymus, and can thus receive an ICOS signal in peripheral lymphoid organs, which might allow them to inactivate FOXO1 and further differentiate into Tfr cells.

The mechanism by which FOXO1 affects Tfh differentiation appears to include its role in the regulation of *Icos* and *Bcl6*, but in addition, other transcription factors that have been implicated in Tfh differentiation. BATF is required for Tfh differentiation and appears to directly control *Bcl6* (Betz et al., 2010; Ise et al., 2011). Within a 35 kb region of the genome that includes only the *Batf* gene, there is a single and very strong FOXO1 peak (rank 411 of 4333), and this peak is located within 100 bp upstream of the *Batf* TSS (Figure S5). Similarly, IRF4 is required for Tfh differentiation (Bollig et al., 2012), and a FOXO1 binding site was detected 1,200 bp upstream of the *Irf4* TSS, and three peaks were detected 37 kb, 43 kb, and 83 kb downstream (rank 1578, 359, 2050 of 4333). On the other hand, other genes important for Tfh differentiation such as *Id3* and *Ascl2* have no proximal FOXO1 binding sites (Miyazaki et al., 2011; Liu et al.,

2014). With the strong caveat that enhancers can be located up to 1 Mb away from the transcription start site (Smallwood and Ren, 2013), the experiments suggest that FOXO1 plays a role in directly regulating a part of the program of gene expression important for Tfh differentiation.

Tfh cells are known to have altered expression of homing molecules that directly control their localization into the B cell follicles. In addition to increased CXCR5 expression, Tfh cells have been shown to have increased expression of CXCR4 but reduced expression of CCR7, CD62L, PSGL1 (encoded by *Selplg*), and EBI2 (encoded by *Gpr183*) (Estes et al., 2004; Hardtke et al., 2005; Poholek et al., 2010; Kroenke et al., 2012). In accord, *Foxo1*^{KO} cells displayed increased expression of CXCR5 and CXCR4 in comparison with WT Tfh cells, but PSGL1 and CD62L expression was decreased day 4 post immunization. FOXO1 has also been shown to upregulate expression of CCR7 through its control of KLF2 expression. These results raise the possibility that loss of FOXO1 might increase Tfh differentiation by controlling expression of these homing molecules consistent with FOXO1 binding sites located proximal to *Cxcr5*, *Cxcr4*, *Ccr7*, *Selplg*, and *Gpr183*.

We propose that the presence or absence of FOXO1 in the landscape of promoters and enhancers found at early stages of T cell activation is a key step in determining the progression of differentiation that ultimately gives rise to one or more functional T helper cell subsets. An implication of this work is that endocrine signaling known to inactivate FOXO1 in liver, muscle, and fat might do so as well in T cells, and thus the immune response to an infectious agent might be skewed depending upon the physiological condition of the host.

EXPERIMENTAL PROCEDURES

Mice

Mice were maintained in a specific-pathogen free vivarium. All experiments were carried out in accordance to the Institutional Animal Care and Use Committee of University of California, San Diego. *Foxo1*$^{f/f}$, *Foxo1*$^{f/f}$*Cd4Cre* (*Foxo1*^{TKO}), *Foxo1*$^{f/f}$*Cd4Cre* OTII, and *Foxo1*$^{f/f}$*Rosa26*$^{Cre-ERT2}$ (*Foxo1*^{KO}) mice of mixed C57BL/6 and FVB genetic backgrounds have been previously described (Kerdiles et al., 2010). For other experiments, *Foxo1*$^{f/f}$ mice were backcrossed to C57BL/6 (Jackson) for at least 13 generations and then crossed to *Rosa26*$^{Cre-ERT2}$, which had also been backcrossed to C57BL/6 for 10 generations, and OTII to generate backcrossed *Foxo1*$^{f/f}$*Rosa26*$^{Cre-ERT2}$ and *Foxo1*$^{f/f}$ *Rosa26*$^{Cre-ERT2}$ OTII mice. For additional controls, OTII mice were crossed to CD45.1, or *Rosa26*$^{Cre-ERT2}$ mice as indicated. *Rosa26-hFoxo1*^{AAA} (*Foxo1*^{AAA}) (Ouyang et al., 2012) were bred to OT-II mice. Unless otherwise indicated, CD45.1 mice were used as hosts for adoptive transfer experiments. CD45.1 mice were purchased from Jackson Laboratories and maintained in our colony. *Foxo1*$^{f/f}$*Cd4Cre* mice were crossed to B6.129P2-ICOStm1Mak/J (*Icos*$^{-/-}$) mice from Jackson Laboratories. *Icosl*$^{-/-}$ host mice were purchased from Jackson Laboratories and maintained at La Jolla Institute for Allergy and Immunology. Bone-marrow chimera experiments were carried out at the University of Washington. The FOXO1-EGFP knock-in mice were generated at Taconic as described in Supplemental Information.

Adoptive Transfer Experiments

For in vivo Tfh cell experiments in which mice were immunized, OTII cells were enriched by negative magnetic selection for naive CD4 (CD69^{-}CD25^{-}CD4^{+}) cells and 0.1 to 0.5 × 10^6 OTII cells were adoptively transferred into CD45.1 hosts unless other indicated. Approximately 2 to 12 hr later, mice were immunized intraperitoneally (i.p.) with 0.1 mg of OVA in 200 μl of Sigma Adjuvant System (Sigma) as per manufacturer's instructions. The phenotype of transferred splenocytes at indicated days post immunization was determined. For experiments plus or minus inhibitory ICOSL-specific antibody (Clone: HK5.3, BioXCell) 100 μg of anti-ICOSL or isotype control were injected intravenously (i.v.) and an additional 100 μg were injected i.p. immediately prior to immunizations. An additional 100 μg of the appropriate antibody was injected i.p. 2 dpi. Where indicated, mice were infected with 10 × 10^6 cfu of ΔActA-Lm-OVA i.v.

For VSV-OVA co-transfer experiments, 10,000 cells of each WT OTII (CD45.1.2) and *Foxo1*^{KO} OTII (CD45.2) cells were transferred into the same host mice and the next day mice were infected with 10^5 pfu of VSV-OVA. Phenotype of transferred cells was determined 6–7 days post infection by flow cytometry.

In Vitro ICOS Signaling Experiments

To study ICOS signaling, we activated and restimulated cells with anti-ICOS similarly to previously described ICOS restimulation conditions (Rolf et al., 2010a). Briefly, WT or FOXO1-GFP naive CD4 (CD69^{-}CD25^{-}CD4^{+}) T cells were purified by negative depletion and activated with anti-CD3 (2C11), 1 μg/ml anti-CD28 plus or minus 10 μg/ml anti-IFN-γ, 10 μg/ml anti-IL-4, 50 ng/ml IL-6, and 10 ng/ml IL-21 (iTfh conditions) in RP10 for 48 hr. After 48 hr, the cells were rested in RP10 for 24 hr. Following the rest, the cells were restimulated with soluble anti-CD3 0.5 μg/ml, goat anti-hamster 20 μg/ml (Vector Labs, Burlingame, CA) with or without stimulatory 2 μg/ml anti-ICOS (Clone: C398.4A, eBioscience). To determine whether ICOS signaling inactivated, we collected FOXO1 cells at 30 m or 24 hr post restimulation and analyzed FOXO1-GFP compared to DRAQ5 staining using AMNIS ImageStream and BD LSR Fortessa analysis. To determine whether ICOS signaling through FOXO1 might be involved in ICOS upregulation of BCL6, we left WT or *Foxo1*^{KO} CD4 T cells in culture for 24 hr post restimulation and analyzed expression of Tfh markers by flow cytometry.

Imaging Flow Cytometry

FOXO1-GFP localization was determined using the 60× objective on ImageStreamX MkII (Amnis/EMD Millipore). FOXO1-GFP signal was compared to the nuclear mask generated using signal from DRAQ5 (Cell Signaling). Data was analyzed with IDEAS software including the nuclear localization wizard. To determine percent of cells with cytoplasmic FOXO1-GFP, we gated cells with a similarity score from FOXO1-GFP and DRAQ5 less than the similarity score that was determined by visual examination of images to represent cells is which FOXO1-GFP was excluded from the nucleus. The nuclear intensity of FOXO1-GFP reflects the amount of FOXO1-GFP within the DRAQ5 nuclear mask.

Generation, Infection, and Analysis of Mixed Bone-Marrow Chimeras

Bone-marrow cells were harvested from femurs, tibias, and humeri. T cells were depleted from bone-marrow cell suspensions with anti-Thy1.2 (30-H12, eBioscience) and low-toxicity rabbit complement (Cedarlane Laboratories). CD45.1^{+} wild-type bone-marrow cells were mixed with 4-fold excess CD45.2^{+} Foxo1TKO bone-marrow cells. 5–10^6 total bone-marrow cells were injected into lethally irradiated (10 Gy) CD45.1.2^{+} hosts. Eight weeks later, chimerism was assessed by flow cytometry and mice were injected intravenously with 10^7 actA deficient *Listeria monocytogenes* (Lm) bacteria engineered to secrete a fusion protein containing an immunogenic peptide (Lm-2W) (Ertelt et al., 2009). Nine days later, mice were sacrificed, spleen and lymph node cells were harvested, and lymphocytes were stained for 1 hr at room temperature with LLOp:I-A^{b}-streptavidin-allophycocyanin tetramers and 2 mg of phycoerythrin-conjugated antibody specific for CXCR5 (2G8; Becton Dickinson). Samples were then enriched for bead-bound cells on magnetized columns (Moon et al., 2007). Cells were then analyzed by flow cytometry.

Statistical Analyses

Unless otherwise indicated two-tailed, unpaired Student t tests were used to determine statistical significance. * $p < 0.05$, ** $p < 0.01$, *** $p < 0.001$.

SUPPLEMENTAL INFORMATION

Supplemental Information includes five figures and Supplemental Experimental Procedures and can be found with this article online at http://dx.doi.org/10.1016/j.immuni.2015.01.017.

ACKNOWLEDGMENTS

We appreciate advice on retroviral transductions provided by J. Scott-Browne (LIAI) and K. Omilusik (UCSD). We thank Shane Crotty (LIAI) for critically reviewing an early version of this manuscript and providing *Icosl*$^{-/-}$ mice. We thank J. Yang, Y.S. Choi, T. Escobar (LIAI), and B.M. Wellisch (UCSD) for technical assistance. We thank Y. Altman (SBMRI) for technical expertise on the Amnis ImageStream X MkII at the Sanford-Burnham Institute. We thank BD Biosciences for the anti-BCL6 (clone 2G8). E.L.S. was supported by an (K12) Institutional Research and Academic Career Development Award NIGMS (L. Brunton, Director) and a K01 award 1K01DK095008. This work was supported by NIH grants RO1AI073885 (S.M.H.) and RO1AI067545 (A.W.G.).

Received: August 5, 2013
Revised: November 13, 2014
Accepted: December 3, 2014
Published: February 17, 2015

REFERENCES

Bauquet, A.T., Jin, H., Paterson, A.M., Mitsdoerffer, M., Ho, I.C., Sharpe, A.H., and Kuchroo, V.K. (2009). The costimulatory molecule ICOS regulates the expression of c-Maf and IL-21 in the development of follicular T helper cells and TH-17 cells. Nat. Immunol. *10*, 167–175.

Betz, B.C., Jordan-Williams, K.L., Wang, C., Kang, S.G., Liao, J., Logan, M.R., Kim, C.H., and Taparowsky, E.J. (2010). Batf coordinates multiple aspects of B and T cell function required for normal antibody responses. J. Exp. Med. *207*, 933–942.

Bollig, N., Brüstle, A., Kellner, K., Ackermann, W., Abass, E., Raifer, H., Camara, B., Brendel, C., Giel, G., Bothur, E., et al. (2012). Transcription factor IRF4 determines germinal center formation through follicular T-helper cell differentiation. Proc. Natl. Acad. Sci. USA *109*, 8664–8669.

Calnan, D.R., and Brunet, A. (2008). The FoxO code. Oncogene *27*, 2276–2288.

Choi, Y.S., Kageyama, R., Eto, D., Escobar, T.C., Johnston, R.J., Monticelli, L., Lao, C., and Crotty, S. (2011). ICOS receptor instructs T follicular helper cell versus effector cell differentiation via induction of the transcriptional repressor Bcl6. Immunity *34*, 932–946.

Choi, Y.S., Yang, J.A., and Crotty, S. (2013). Dynamic regulation of Bcl6 in follicular helper CD4 T (Tfh) cells. Curr. Opin. Immunol. *25*, 366–372.

Chung, Y., Tanaka, S., Chu, F., Nurieva, R.I., Martinez, G.J., Rawal, S., Wang, Y.H., Lim, H., Reynolds, J.M., Zhou, X.H., et al. (2011). Follicular regulatory T cells expressing Foxp3 and Bcl-6 suppress germinal center reactions. Nat. Med. *17*, 983–988.

Crotty, S. (2014). T follicular helper cell differentiation, function, and roles in disease. Immunity *41*, 529–542.

Ertelt, J.M., Rowe, J.H., Johanns, T.M., Lai, J.C., McLachlan, J.B., and Way, S.S. (2009). Selective priming and expansion of antigen-specific Foxp3- CD4+ T cells during Listeria monocytogenes infection. J. Immunol. *182*, 3032–3038.

Estes, J.D., Thacker, T.C., Hampton, D.L., Kell, S.A., Keele, B.F., Palenske, E.A., Druey, K.M., and Burton, G.F. (2004). Follicular dendritic cell regulation of CXCR4-mediated germinal center CD4 T cell migration. J. Immunol. *173*, 6169–6178.

Fabre, S., Lang, V., Harriague, J., Jobart, A., Unterman, T.G., Trautmann, A., and Bismuth, G. (2005). Stable activation of phosphatidylinositol 3-kinase in the T cell immunological synapse stimulates Akt signaling to FoxO1 nuclear exclusion and cell growth control. J. Immunol. *174*, 4161–4171.

Fabre, S., Carrette, F., Chen, J., Lang, V., Semichon, M., Denoyelle, C., Lazar, V., Cagnard, N., Dubart-Kupperschmitt, A., Mangeney, M., et al. (2008). FOXO1 regulates L-Selectin and a network of human T cell homing molecules downstream of phosphatidylinositol 3-kinase. J. Immunol. *181*, 2980–2989.

Franko, J.L., and Levine, A.D. (2009). Antigen-independent adhesion and cell spreading by inducible costimulator engagement inhibits T cell migration in a PI-3K-dependent manner. J. Leukoc. Biol. *85*, 526–538.

Fu, Z., and Tindall, D.J. (2008). FOXOs, cancer and regulation of apoptosis. Oncogene *27*, 2312–2319.

Gigoux, M., Shang, J., Pak, Y., Xu, M., Choe, J., Mak, T.W., and Suh, W.K. (2009). Inducible costimulator promotes helper T-cell differentiation through phosphoinositide 3-kinase. Proc. Natl. Acad. Sci. USA *106*, 20371–20376.

Hardtke, S., Ohl, L., and Förster, R. (2005). Balanced expression of CXCR5 and CCR7 on follicular T helper cells determines their transient positioning to lymph node follicles and is essential for efficient B-cell help. Blood *106*, 1924–1931.

Hedrick, S.M. (2009). The cunning little vixen: Foxo and the cycle of life and death. Nat. Immunol. *10*, 1057–1063.

Hedrick, S.M., Hess Michelini, R., Doedens, A.L., Goldrath, A.W., and Stone, E.L. (2012). FOXO transcription factors throughout T cell biology. Nat. Rev. Immunol. *12*, 649–661.

Hess Michelini, R., Doedens, A.L., Goldrath, A.W., and Hedrick, S.M. (2013). Differentiation of CD8 memory T cells depends on Foxo1. J. Exp. Med. *210*, 1189–1200.

Ise, W., Kohyama, M., Schraml, B.U., Zhang, T., Schwer, B., Basu, U., Alt, F.W., Tang, J., Oltz, E.M., Murphy, T.L., and Murphy, K.M. (2011). The transcription factor BATF controls the global regulators of class-switch recombination in both B cells and T cells. Nat. Immunol. *12*, 536–543.

Kerdiles, Y.M., Beisner, D.R., Tinoco, R., Dejean, A.S., Castrillon, D.H., DePinho, R.A., and Hedrick, S.M. (2009). Foxo1 links homing and survival of naive T cells by regulating L-selectin, CCR7 and interleukin 7 receptor. Nat. Immunol. *10*, 176–184.

Kerdiles, Y.M., Stone, E.L., Beisner, D.R., McGargill, M.A., Ch'en, I.L., Stockmann, C., Katayama, C.D., and Hedrick, S.M. (2010). Foxo transcription factors control regulatory T cell development and function. Immunity *33*, 890–904.

Kroenke, M.A., Eto, D., Locci, M., Cho, M., Davidson, T., Haddad, E.K., and Crotty, S. (2012). Bcl6 and Maf cooperate to instruct human follicular helper CD4 T cell differentiation. J. Immunol. *188*, 3734–3744.

Linterman, M.A., Pierson, W., Lee, S.K., Kallies, A., Kawamoto, S., Rayner, T.F., Srivastava, M., Divekar, D.P., Beaton, L., Hogan, J.J., et al. (2011). Foxp3+ follicular regulatory T cells control the germinal center response. Nat. Med. *17*, 975–982.

Liu, X., Nurieva, R.I., and Dong, C. (2013). Transcriptional regulation of follicular T-helper (Tfh) cells. Immunol. Rev. *252*, 139–145.

Liu, X., Chen, X., Zhong, B., Wang, A., Wang, X., Chu, F., Nurieva, R.I., Yan, X., Chen, P., van der Flier, L.G., et al. (2014). Transcription factor achaete-scute homologue 2 initiates follicular T-helper-cell development. Nature *507*, 513–518.

Migliazza, A., Martinotti, S., Chen, W., Fusco, C., Ye, B.H., Knowles, D.M., Offit, K., Chaganti, R.S., and Dalla-Favera, R. (1995). Frequent somatic hypermutation of the 5′ noncoding region of the BCL6 gene in B-cell lymphoma. Proc. Natl. Acad. Sci. USA *92*, 12520–12524.

Miyazaki, M., Rivera, R.R., Miyazaki, K., Lin, Y.C., Agata, Y., and Murre, C. (2011). The opposing roles of the transcription factor E2A and its antagonist Id3 that orchestrate and enforce the naive fate of T cells. Nat. Immunol. *12*, 992–1001.

Moon, J.J., Chu, H.H., Pepper, M., McSorley, S.J., Jameson, S.C., Kedl, R.M., and Jenkins, M.K. (2007). Naive CD4(+) T cell frequency varies for different epitopes and predicts repertoire diversity and response magnitude. Immunity *27*, 203–213.

Odegard, J.M., Marks, B.R., DiPlacido, L.D., Poholek, A.C., Kono, D.H., Dong, C., Flavell, R.A., and Craft, J. (2008). ICOS-dependent extrafollicular helper T cells elicit IgG production via IL-21 in systemic autoimmunity. J. Exp. Med. *205*, 2873–2886.

Oestreich, K.J., Mohn, S.E., and Weinmann, A.S. (2012). Molecular mechanisms that control the expression and activity of Bcl-6 in TH1 cells to regulate flexibility with a TFH-like gene profile. Nat. Immunol. *13*, 405–411.

Ouyang, W., and Li, M.O. (2011). Foxo: in command of T lymphocyte homeostasis and tolerance. Trends Immunol. *32*, 26–33.

Ouyang, W., Beckett, O., Ma, Q., Paik, J.H., DePinho, R.A., and Li, M.O. (2010). Foxo proteins cooperatively control the differentiation of Foxp3+ regulatory T cells. Nat. Immunol. *11*, 618–627.

Ouyang, W., Liao, W., Luo, C.T., Yin, N., Huse, M., Kim, M.V., Peng, M., Chan, P., Ma, Q., Mo, Y., et al. (2012). Novel Foxo1-dependent transcriptional programs control T(reg) cell function. Nature *491*, 554–559.

Pellicano, F., and Holyoake, T.L. (2011). Assembling defenses against therapy-resistant leukemic stem cells: Bcl6 joins the ranks. J. Exp. Med. *208*, 2155–2158.

Pepper, M., Pagán, A.J., Igyártó, B.Z., Taylor, J.J., and Jenkins, M.K. (2011). Opposing signals from the Bcl6 transcription factor and the interleukin-2 receptor generate T helper 1 central and effector memory cells. Immunity *35*, 583–595.

Poholek, A.C., Hansen, K., Hernandez, S.G., Eto, D., Chandele, A., Weinstein, J.S., Dong, X., Odegard, J.M., Kaech, S.M., Dent, A.L., et al. (2010). In Vivo Regulation of Bcl6 and T Follicular Helper Cell Development. J Immunol. *185*, 313–326.

Qi, H., Liu, D., Ma, W., Wang, Y., and Yan, H. (2014). Bcl-6 controlled TFH polarization and memory: the known unknowns. Curr. Opin. Immunol. *28*, 34–41.

Ramiscal, R.R., and Vinuesa, C.G. (2013). T-cell subsets in the germinal center. Immunol. Rev. *252*, 146–155.

Rolf, J., Bell, S.E., Kovesdi, D., Janas, M.L., Soond, D.R., Webb, L.M., Santinelli, S., Saunders, T., Hebeis, B., Killeen, N., et al. (2010a). Phosphoinositide 3-kinase activity in T cells regulates the magnitude of the germinal center reaction. J. Immunol. *185*, 4042–4052.

Rolf, J., Fairfax, K., and Turner, M. (2010b). Signaling pathways in T follicular helper cells. J. Immunol. *184*, 6563–6568.

Sage, P.T., Francisco, L.M., Carman, C.V., and Sharpe, A.H. (2013). The receptor PD-1 controls follicular regulatory T cells in the lymph nodes and blood. Nat. Immunol. *14*, 152–161.

Seo, Y.B., Im, S.J., Namkoong, H., Kim, S.W., Choi, Y.W., Kang, M.C., Lim, H.S., Jin, H.T., Yang, S.H., Cho, M.L., et al. (2014). Crucial role of IL-7 in the development of T follicular helper cells as well as the induction of humoral immunity. J. Virol. *88*, 8998–9009.

Silver, D.P., and Livingston, D.M. (2001). Self-excising retroviral vectors encoding the Cre recombinase overcome Cre-mediated cellular toxicity. Mol. Cell *8*, 233–243.

Smallwood, A., and Ren, B. (2013). Genome organization and long-range regulation of gene expression by enhancers. Curr. Opin. Cell Biol. *25*, 387–394.

Stahl, M., Dijkers, P.F., Kops, G.J., Lens, S.M., Coffer, P.J., Burgering, B.M., and Medema, R.H. (2002). The forkhead transcription factor FoxO regulates transcription of p27Kip1 and Bim in response to IL-2. J. Immunol. *168*, 5024–5031.

Surh, C.D., and Sprent, J. (2008). Homeostasis of naive and memory T cells. Immunity *29*, 848–862.

Ueno, H., Banchereau, J., and Vinuesa, C.G. (2015). Pathophysiology of T follicular helper cells in humans and mice. Nat. Immunol. *16*, 142–152.

Vinuesa, C.G., Tangye, S.G., Moser, B., and Mackay, C.R. (2005). Follicular B helper T cells in antibody responses and autoimmunity. Nat. Rev. Immunol. *5*, 853–865.

Walker, S.R., Nelson, E.A., Yeh, J.E., Pinello, L., Yuan, G.C., and Frank, D.A. (2013). STAT5 outcompetes STAT3 to regulate the expression of the oncogenic transcriptional modulator BCL6. Mol. Cell. Biol. *33*, 2879–2890.

Wang, H., Geng, J., Wen, X., Bi, E., Kossenkov, A.V., Wolf, A.I., Tas, J., Choi, Y.S., Takata, H., Day, T.J., et al. (2014). The transcription factor Foxp1 is a critical negative regulator of the differentiation of follicular helper T cells. Nat. Immunol. *15*, 667–675.

Xiao, N., Eto, D., Elly, C., Peng, G., Crotty, S., and Liu, Y.C. (2014). The E3 ubiquitin ligase Itch is required for the differentiation of follicular helper T cells. Nat. Immunol. *15*, 657–666.

Xu, H., Li, X., Liu, D., Li, J., Zhang, X., Chen, X., Hou, S., Peng, L., Xu, C., Liu, W., et al. (2013). Follicular T-helper cell recruitment governed by bystander B cells and ICOS-driven motility. Nature *496*, 523–527.

Yu, Q., Erman, B., Park, J.H., Feigenbaum, L., and Singer, A. (2004). IL-7 receptor signals inhibit expression of transcription factors TCF-1, LEF-1, and RORgammat: impact on thymocyte development. J. Exp. Med. *200*, 797–803.

Immunity

Supplemental Information

ICOS Coreceptor Signaling Inactivates the Transcription Factor FOXO1 to Promote Tfh Cell Differentiation

Erica L. Stone, Marion Pepper, Carol D. Katayama, Yann M. Kerdiles, Chen-Yen Lai, Elizabeth Emslie, Yin C. Lin, Edward Yang, Ananda W. Goldrath, Ming O. Li, Doreen A. Cantrell, Stephen M. Hedrick

Figure S1; Stone et al.

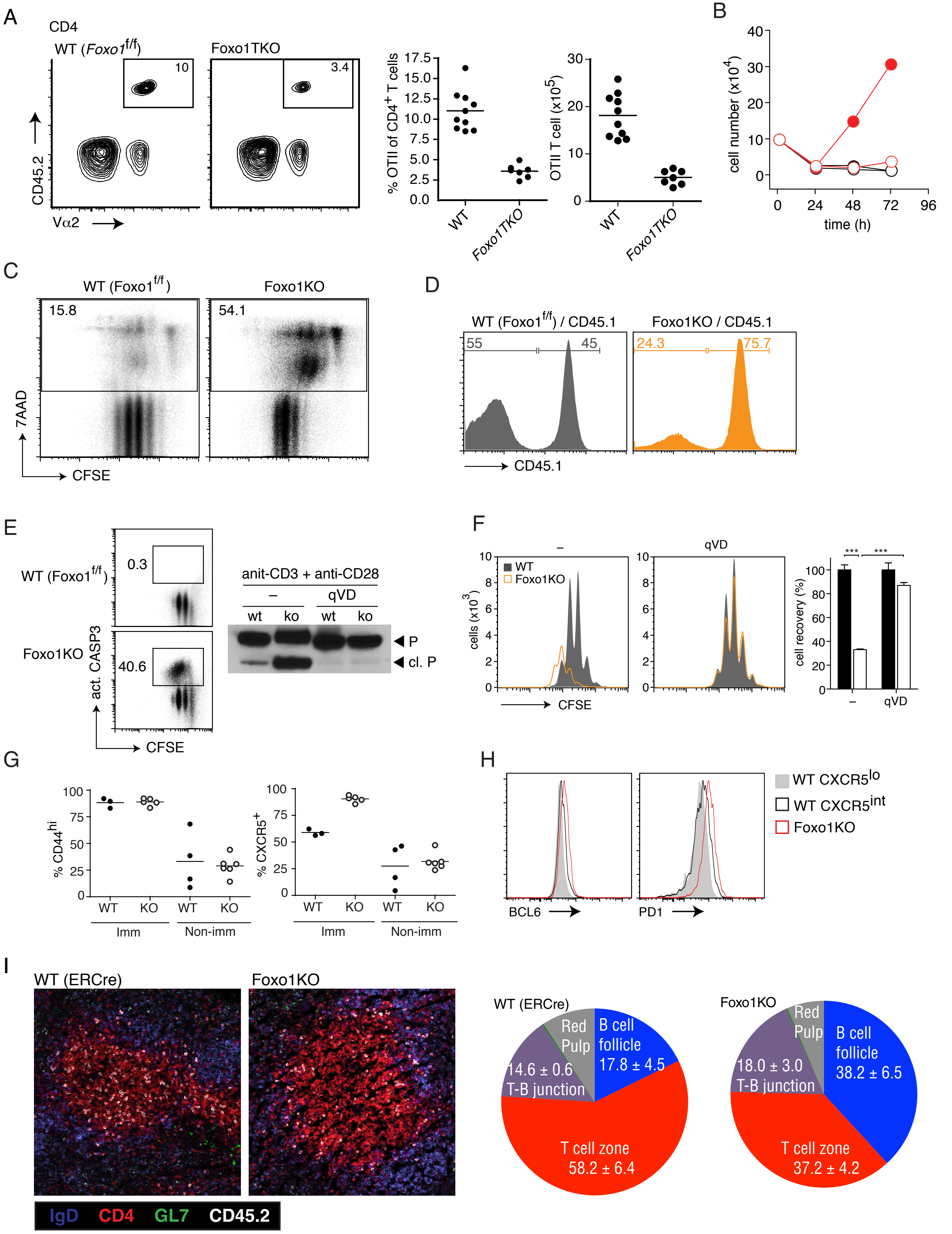

Figure S2, Stone et al.

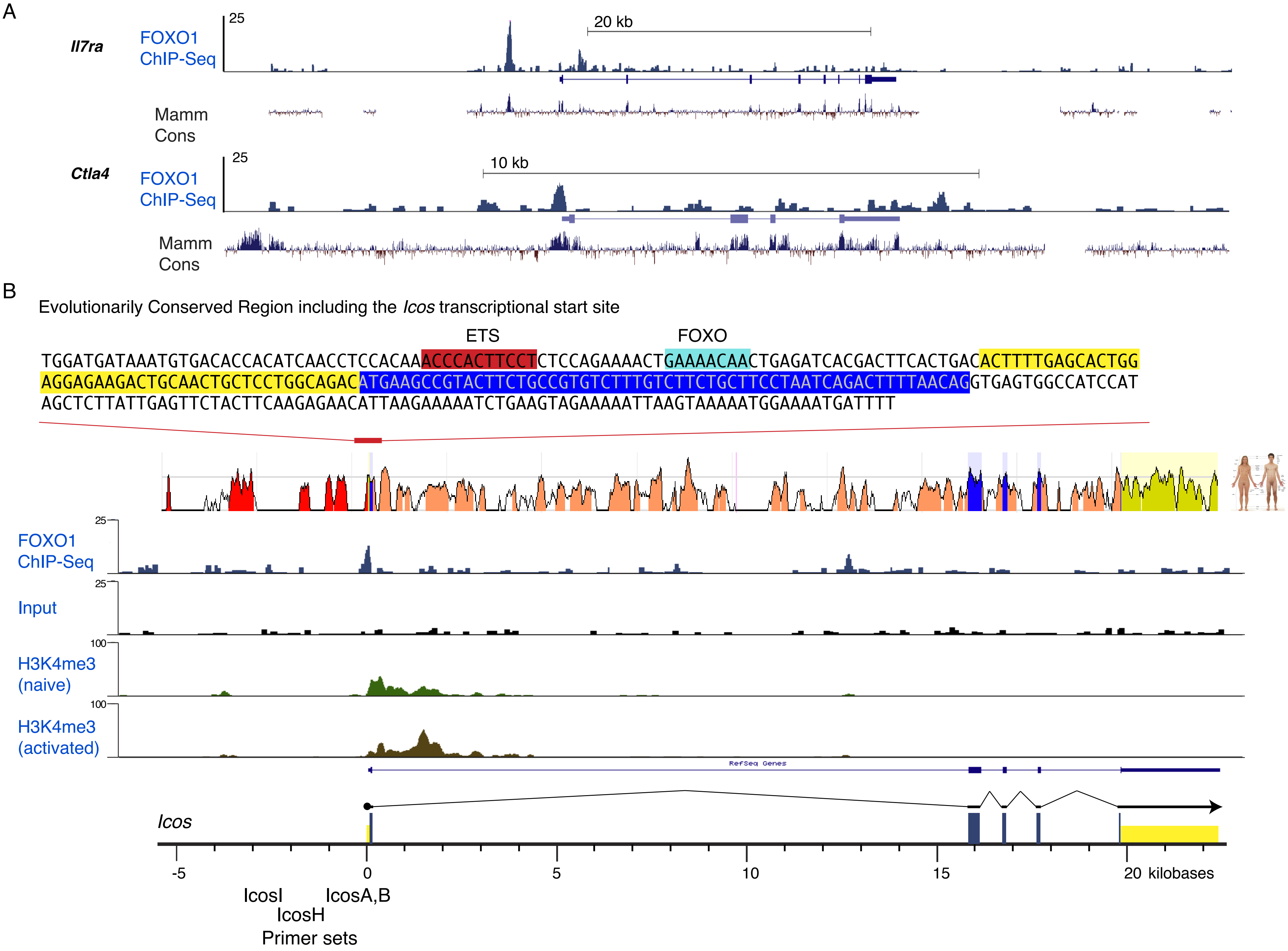

Figure S3, Stone et al.

A

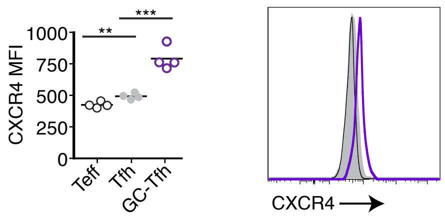

B

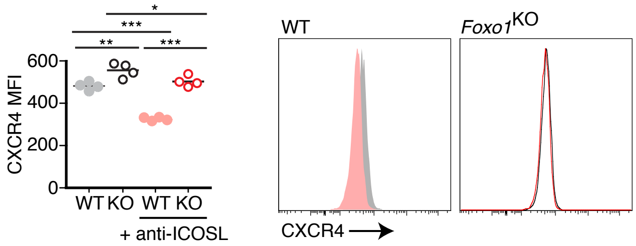

Figure S4, Stone et al.

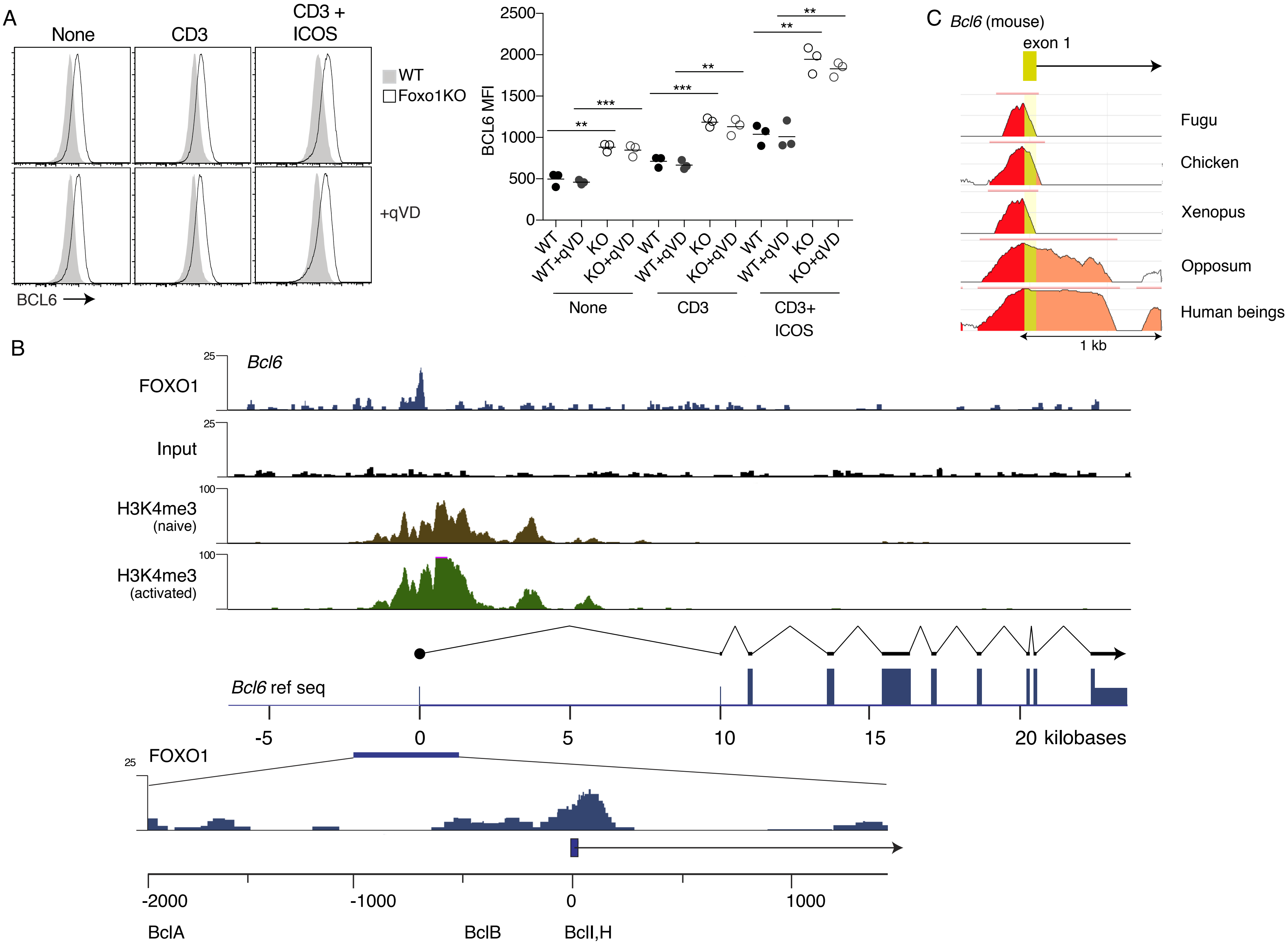

Figure S5, Stone et al.

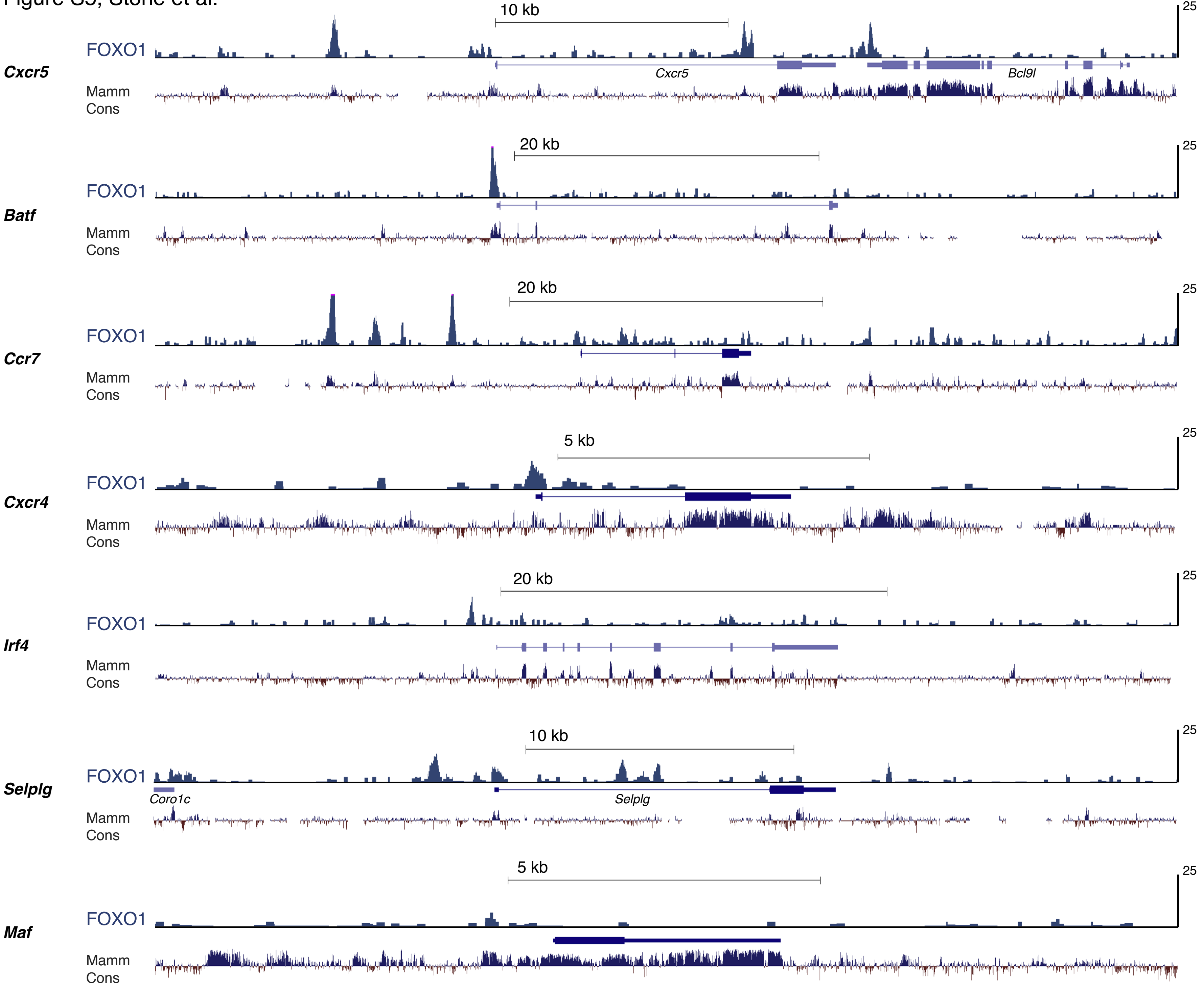

SUPPLEMENTAL FIGURE LEGENDS

Figure S1, related to Figure 1. FOXO1 is required to prevent activated CD4 T cell death

(A) Decreased accummulation of *Foxo1*^{TKO} T cells *in vivo*. OTII T cells were transferred into CD45.1 hosts, and immunized with OVA and adjuvant. At d 4, WT or *Foxo1*^{TKO} OTII CD4+ cells were analyzed for accumulation by gating for CD45.2^{+}Vα2^{+} T cells. Each dot represents a single host mouse. One of two representative experiments.

(B-F) Activated CD4 *Foxo1*^{KO} T cells die by apoptosis. LN naïve CD4 T cells (CD4^{+} CD69$^{–}$ CD25$^{–}$) were isolated from tamoxifen-treated WT (*Foxo1*$^{f/f}$, filled circles) and *Foxo1*^{KO} (open circles) and tested as shown. (B) Decreased accumulation of *Foxo1*^{KO} CD4 T cells *in vitro*. Enumeration of purified naïve LN CD4 T cells stimulated with anti-CD3 in presence (red circles) or absence (black circles) of soluble anti-CD28 (mean + s.d. of triplicate cultures, one representative experiment out of three). (C) *Foxo1*^{KO} CD4 T cells progress normally through cell division but die at a high rate in the first rounds of division. Representative FACS profile of CFSE dilution vs. 7AAD from naïve LN CD4 T cells stimulated for 2 days with anti-CD3 and anti-CD28 (one representative experiment out of three). (D) Loss of *Foxo1*^{KO} CD4 T cells is cell-intrinsic. Relative cell recovery of anti-CD3 plus anti-CD28 stimulated naïve LN CD4 T cells co-cultured with naïve LN CD4 T cells from WT CD45.1 mice for 3 days (one representative experiment out of two). (E) Stimulation of *Foxo1*^{KO} CD4 T cells provokes active Caspase 3. Representative FACS profile of naïve LN CD4 T cells stimulated for 2 d with anti-CD3/anti-CD28 (one representative experiment out of two) (Left). Activated *Foxo1*^{KO} CD4 T cells display cleaved PARP. Western blot analysis of PARP cleavage in naïve LN CD4 T cells stimulated for 2 days with anti-CD3 plus anti-CD28 in the presence or absence of the pan-caspase inhibitor Q-VD-OPH (qVD) (one representative experiment out of two) (Right). (F) qVD rescues the accummulation of *Foxo1*^{KO} CD4 T cells. Naïve, CFSE-labeled LN CD4 T cells were stimulated for 3 days with anti-CD3 plus anti-CD28 in the presence or absence of the pan-caspase inhibitor qVD (one representative experiment out of two). The results were expressed as cell recovery from a timed-acquisition, such that the area under each curve is representative of the total accumulation of cells (mean + s.d.). Data is from experimental replicates.

(G) Activation and presence of Tfh phenotype in *Foxo1*^{KO} CD4 T cells is antigen dependent. Graphs show percentage of CD44^{hi} (Left) or CXCR5^{+} (Right) of CD4^{+}CD45.2^{+}Vα2^{+} WT or *Foxo1*^{KO} cells 4 days post immunization or non-immunized controls. (H) Total *Foxo1*^{KO} CD4 T cells express higher levels of Tfh markers than WT Tfh cells. Histograms show expression of BCL6 or PD1 d 4 post immunization within the WT (CD4^{+}CD45.2^{+}Vα2^{+}) CXCR5^{lo} or CXCR5^{int} population along with the total *Foxo1*^{KO} (CD4^{+}CD45.2^{+}Vα2^{+}) population for comparison. (I) T cells lacking FOXO1 localize to the follicular border. Immunofluorescence of spleens d 4 post immunization (Left). Pie charts show proportion of OTII cells (mean percentage +/- s.e.m.) located in each region determined by counting

one representative image in a blinded fashion from each of four mice per a donor genotype pooled from multiple experiments (Right). P-value is less than 0.05 between WT and *Foxo1*^{KO} in the T cell zone and between WT and *Foxo1*^{KO} in the B cell follicle.

Figure S2, related to Figure 2. Evolutionarily conserved FOXO1 binding sites within the *Il7ra, Ctla4* and *Icos* loci

(A) We have previously shown by ChIP that FOXO1 binds to an enhancer 3.3 kb upstream of the *Il7ra* TSS and within the promoter of the *Ctla4* gene (Kerdiles et al., 2009; Kerdiles et al., 2010). FOXO1 binding to the *Il7ra* and *Ctla4* locus as determined by ChIPSeq on naïve T cells is shown. (B) The *Icos* locus. Evolutionarily Conserved Regions (ECRs) in the mouse *Icos* gene were compared with human *ICOS*. The Ref Seq data was used to generate a map of mouse *Icos*, and the ECRs taken from decode.org are shown for comparison. Red ECRs are intergenic, yellow indicates untranslated transcriptional regions, and blue denotes exons. The ECR flanking the transcriptional start site was exploded, and it was predicted to have consensus ETS (red) and FOXO (cyan) binding sites. The approximate locations of four primer sets used for ChIP are shown (A and B are overlapping). FOXO1 ChIP-seq analysis at *Icos* locus is reshown from Figure 2F for comparison. Primer sequences are as follows: icosA for:CATACATCACCGGGTACTTGC, icosA rev:GCAGTCTTCTCCTCCAGTGC, icosB for:CCATTCATACATCACCGGGTA, icosB rev:CCAGGAGCAGTTGCAGTCTT, icosH for:GAGGCCAGAAGAGGACACTG, icosH rev:GCATGCCTGTAACCACAGAA, icosI for:AAGCATCCACCCTACAAACG, icosI rev:TGGATGTGAGAATGGAAGGA.

Figure S3, related to Figure 3. *Foxo1*^{KO} OTII cells are less dependent on ICOS for CXCR4 upregulation

Graphs show CXCR4 MFI d 4 post immunization of (A) WT CXCR5^{lo}, CXCR5^{int}, or CXCR5^{hi} OTII cells or (B) total WT or *Foxo1*^{KO} OTII cells from host mice treated with anti-ICOSL or isotype control. Data is from one experiment with at least four host mice per a genotype and condition.

Figure S4, related to Figure 5. Evolutionarily conserved FOXO1 binding site at the *Bcl6* locus

(A) WT (*Foxo1*$^{f/f}$) or *Foxo1*^{KO} naïve CD4 cells were activated as in Figure 5A in the presence or absence of the pan-caspase inhibitor Q-VD-OH (qVD) and BCL6 expression was determined by flow cytometry. Data is representative of one experiment with three mice per a genotype. (B) The *Bcl6* locus. ChIP-Seq data from the Bcl6 locus is shown for FOXO1, and H3K4m3 marks on both naïve and activated T cells. FOXO1 ChIP-Seq analysis at *Bcl6* locus is reshown from Figure 5D for comparison. The Ref Seq data was used to generate a map of mouse *Bcl6*. And the region surrounding the transcription start site is exploded to pinpoint the FOXO1 binding peak. The

approximate locations of four primer sets used for ChIP are shown. BclA is a negative control at approximately -2 kbp, and BclB corresponds to a FOXO1 binding site previously identified (Oestreich et al., 2012). Primer sequences are as follows: bclA for:GTACTCCAACAACAGCACAGC, bclA rev:GTGGCTCGTTAAATCACAGAGG, bclH for:GAGCAATGGTAAAGCCCG, bclH rev:CAACAGCAATAATCACCTGG, bclI for:CGCTGCTCATGATCATTAT, bclI rev:GTATGCGAAAAGCTAGATCCT, IL7Ra for:ACCTCATCAGCCTTTCATGG, IL7Ra rev:ATCCCCTGAGCAAACTAGCA.(C) *Bcl6* ECRs within the region surrounding the first exon compared with: Human beings, Opposum, Xenopus, Chicken, and Fugu. The pink line above each element of the figure indicates conservation of at least 100 bp with 70% or more similarity. We note the extraordinary conservation of the first non-coding exon and the 5' end of the first intron.

Figure S5, related to Figure 6. ChIP-seq reveals FOXO1 binding to key Tfh genes.

The *Cxcr5*, *Batf*, *Ccr7*, *Cxcr4*, *Irf4*, *Selplg* and *Maf* loci are shown for FOXO1-specific ChIP-Seq and mammalian sequence conservation (UCSC genome browser).

SUPPLEMENTAL EXPERIMENTAL PROCEDURES, related to Experimental procedures.

Flow cytometry Generation of FOXO1-EGFP knock-in mice

The FOXO1-EGFP knock-in mice were generated at Taconic. Briefly, the *EGFP* sequence was inserted between the codon coding for the last amino acid of FOXO1 and the termination codon in exon 2 of the *Foxo1* gene which allows for translation of a FOXO1-EGFP fusion protein. Furthermore, exon 2 including the *EGFP* sequence was flanked by *loxP* sites allowing conditional deletion. The targeting vector included *FRT* sites to allow for FLP-mediated removal of *Neo*^{r}. The targeting vector was transfected into TaconicArtemis C57BL/6N Tac ES cells and selected for homologous recombination. FLP-mediated removal of selection markers was accomplished by breeding to mice expressing a *Flp transgene*.

Flow cytometry

Fluorochrome-conjugated antibodies for flow cytometry were from BD Biosciences (San Diego, CA), Biolegend (San Diego, CA) or eBioscience (San Diego, CA) unless otherwise indicated. For CXCR5 a teritary staining protocol was used. Rat anti-mouse CXCR5 (Clone 2G8; BD Biosciences) was allowed to bind for 1h at 4 degrees, biotin-labeled goat anti-rat IgG (Jackson ImmunoResearch) was used as the secondary and allowed to bind for 30 min at 4°C. Finally, fluorochrome-conjugated strepavidin was added with a cocktail of the remaining extracellular antibodies. Cells were fixed and permeabilized using the Foxp3 Permeabilization/Fixation kit (eBioscience, San Diego, CA). FOXO1 intracellular staining was done as previously described (Kerdiles et al., 2010). Cells were analyzed using LSR Fortessa or Calibur flow cytometers (Becton Dickinson) unless otherwise indicated. Traditional flow cytometry data were analyzed with FlowJo software (TreeStar).

Immunofluorescence

Spleen sections were prepared for immunofluorescence as previously described (Cheung et al., 2009). Briefly, freshly harvested tissues were fixed with 4% formaldehyde and soaked in sucrose overnight before embedding in OCT and freezing with dry ice. Six-micrometer-thick tissue sections were cut and fixed in paraformaldehyde. Sections were blocked in a solution of 10% BSA, 2.5%

normal goat serum, 2.5% normal donkey serum, and fish scale gelatin. Tissue sections were then incubated with combinations of conjugated or biotinylated antibodies. Sections were mounted using Invitrogen ProLong Gold antifade reagent. Images were taken with an Olympus FV1000 confocal microscope with five laser lines at wavelengths of 405, 458, 488, 515, 543, and 647 nm, using 10× and 20× air objectives. Images were analyzed using ImageJ. One representative image from each spleen was blinded and the proportion of CD45.2^{+} cells in each region of the spleen was determined. For this images were blown up, a grid was placed over the image and then for every CD45.2^{+} cell in the image it was determined by eye if the cell was located in the T cell area, Follicle, T/B boundry, GC or other area. This was done in a blinded fashion.

Retroviral transduction experiments

Retrovirus was generated by transducing plat-E cells with the pHR-MMPCreGFP retroviral vector (Silver and Livingston, 2001). Retroviral transductions were done as previously described with slight modifications (Johnston et al., 2009). Briefly, naive WT or *Foxo1*^{AAA} *OTII* cells were purified and activated with plate bound anti-CD3 and soluble anti-CD28 for 24 h. Cells were transduced with the resulting retrovirus at 24 h and 48 h. Cells were cultured with plate bound anti-CD3 and soluble anti-CD28 and IL-2 from 24 h to 72 h. After 72 h cells were cultured in media supplemented with IL-2 for an additional 3 days after which they were transferred into CD45.1 hosts. Hosts were immunized with OVA plus adjuvant as above 3 days after adoptive transfer.

In vitro death and proliferation experiments

Naive CD4 T cells were isolated by magnetic depletion of cells labeled with biotinylated antibodies to TER119, B220, MHCII, DX5, CD8, CD11B, CD25, and CD69 (eBioscience) and streptavidin-microbeads (Miltenyi Biotec). Where indicated, purified T cells were labeled with CFSE as previously described (D'Souza et al., 2008). To activate T cells, 1 x 10^5 cells/well were cultured in U bottom 96 well plates. Plates were coated with goat anti-Hamster IgG (Vector Labs) followed by anti-CD3 (2C11). Where indicated, cultures were supplemented with soluble anti-CD28 (Biolegend) or 20 μM pan-caspase inhibitor qVD (SM Biochemicals).

ChIP and ChIP-seq

Naïve CD4 T cells were purified as described above. Where indicated 5 x 10^5 cells per a well were activated in 24 well plates coated with anti-CD3 in the presence of anti-CD28 for 72 h. In other experiments cells were stimulated under iTfh conditions. FOXO1 ChIP-seq was done as previously described with minor modifications (Lin et al., 2010). Briefly, 3 x 10^7 T cells were fixed for 5 to 10 minutes at room temperature in 1% formaldehyde then resuspended in lysis buffer. Chromatin was sonicated to an average size of 400-500 base pairs and immunoprecipitated with 8 μg of anti-FOXO1 (clone: H-128, Santa Cruz Biotechnology) or anti-trimethyl-Histone H3 Lys4 (EMD Millipore). Bound chromatin was collected using protein G sepharose and de-crosslinked overnight at 65° C. Following RNaseA and proteinase K treatment, DNA was purified using a Qiaquick PCR Purification kit (Qiagen). For ChIP analysis, QPCR was performed using Fast Start Universal Syber Green master mix from Roche and analyzed on a Stratagene MX3005P real time thermal cycler. Homogeneous PCR products were validated by melting curve analyses.

For ChIP-seq samples were prepared for multiplex sequencing as recommended by Illumina. Following adaptor ligation, the DNAs were size selected (200-300bp) by 8% PAGE and index primers added by PCR. Samples were finally purified by 8% PAGE and precipitated with ethanol. Sequencing was performed by the BIOGEM core at UC San Diego using an Illumina HiSeq 2000 sequencer (50 cycles). Sequences were mapped to the mouse genome mm9 assembly (NCBI) using the Bowtie alignment tool and unique tags were visualized by preparing custom tracks for the UCSC Genome Browser where the total number of tags was normalized to 1 x 10^7. DNA sequence analysis was performed using HOMER software and instructions for analysis can be found at http://biowhat.ucsd.edu/homer/. The mammalian sequence conservation tracks were taken from the UCSC genome browser using the 30-Way Multiz Alignment and Conservation routine. Data has been deposited in the GEO database (Hess Michelini et al., 2013).

ELISA

Mice were bled at 4-6 months of age and anti-dsDNA antibodies of the IgG isotype in sera were determined by anti-mouse dsDNA IgG-specific ELISA kit (Alpha Diagnostic Int). Total sera IgG was

determined using Mouse IgG total Ready-Set-Go! ELISA (eBioscience) as per manufactures instructions.

SUPPLEMENTAL REFERENCES, related to Experimental procedures

Cheung, K.P., Yang, E., and Goldrath, A.W. (2009). Memory-like CD8+ T cells generated during homeostatic proliferation defer to antigen-experienced memory cells. J Immunol *183*, 3364–3372.

D'Souza, W.N., Chang, C.F., Fischer, A.M., Li, M., and Hedrick, S.M. (2008). The Erk2 MAPK regulates CD8 T cell proliferation and survival. J Immunol *181*, 7617–7629.

Johnston, R.J., Poholek, A.C., DiToro, D., Yusuf, I., Eto, D., Barnett, B., Dent, A.L., Craft, J., and Crotty, S. (2009). Bcl6 and Blimp-1 are reciprocal and antagonistic regulators of T follicular helper cell differentiation. Science *325*, 1006–1010.

Lin, Y.C., Jhunjhunwala, S., Benner, C., Heinz, S., Welinder, E., Mansson, R., Sigvardsson, M., Hagman, J., Espinoza, C.A., Dutkowski, J., Ideker, T., Glass, C.K., and Murre, C. (2010). A global network of transcription factors, involving E2A, EBF1 and Foxo1, that orchestrates B cell fate. Nat Immunol *11*, 635–643.
